# Multi-pronged biobehavioural intervention strategies for prevention and control of hypertension: A systematic review of education-based community trials

**DOI:** 10.1177/20503121261444673

**Published:** 2026-05-10

**Authors:** Martins Nweke, Nalini Govender, Bernard Appiah, Julian Pillay

**Affiliations:** 1 Basic Medical Science, Faculty of Health Sciences, Durban University of Technology, South Africa; 2 Research Program on Health Communication and Public Engagement (H-COPE), Department of Public Health, Maxwell School of Citizenship and Public Affairs, Syracuse University, NY, USA

**Keywords:** Hypertension, prevention, control, intervention, community-wide, implementation

## Abstract

**Background:**

Hypertension contributes to preventable morbidity and mortality, especially in low- and middle-income countries with strained healthcare services. Factors such as diet, inactivity, stress, and low treatment adherence worsen this challenge. Community-based education programmes can address hypertension prevention and management. This review thus examines multi-pronged bio-behavioural education interventions outside healthcare facilities.

**Methods:**

Multiple electronic databases were searched for community-based education programmes from inception (1996) to May 2025. Eligible studies evaluated community, workplace, or home-based education programmes that combined lifestyle advice, self-regulation strategies, and self-monitoring. Randomised and non-randomised designs were included if they reported blood pressure outcomes in adults without restriction to baseline hypertension status. Primary outcomes comprised changes in systolic blood pressure (SBP) and diastolic blood pressure (DBP). Data were extracted by a trained assistant, and independently verified by the primary reviewer. Meta-analyses were conducted using random effects models. Risk of bias was assessed using the Cochrane ROBINS-I and RoB-2 tools.

**Results:**

Seventeen studies with 5532 participants met the inclusion criteria. Interventions typically incorporated dietary education, physical activity guidance, stress control techniques, and blood pressure self-monitoring. Programme duration ranged from 4 weeks to 12 months. Pooled results showed a reduction in SBP of 6.42 mmHg (95% CI −9.69 to −3.16) and a reduction in DBP of 3.04 mmHg (95% CI −6.02 to −0.07). Effects were larger in randomised trials and in interventions lasting 6 months or longer. The overall risk of bias was moderate.

**Conclusion:**

Community bio-behavioural education programmes may improve blood pressure and complement pharmacological care, particularly where clinical access is limited. However, very high heterogeneity indicates that effects vary across settings and interventions. Further well-designed studies with longer follow-up are needed to confirm durability and guide implementation.

**Registration:**

The study is registered with PROSPERO, an international register of systematic reviews (ID: CRD420251034159).

## Introduction

Hypertension (HTN) is recognised as the leading preventable cause of cardiovascular disease and premature mortality worldwide.^1,2^ Despite the widespread administration and use of antihypertensive medications, the prevalence of HTN continues to increase, particularly in low- and middle-income countries (LMICs).^1^ As of March 2023, approximately 1.28 billion adults aged 30–79 years are affected by HTN globally, with two-thirds residing in LMICs.^3^ Notably, an estimated 46% of adults with HTN remain unaware of their condition, and only about 21% of those with HTN have achieved control over it.^3^ Moreover, HTN remains the primary cause of stroke and cardiac failure globally.^4,5^ The increasing global burden of HTN is associated with age, unhealthy dietary patterns characterised by high sodium and low potassium intake, and insufficient physical activity.^6^ Globally, changes in HTN prevalence remain disproportionate, with high-income countries experiencing a modest decline in HTN prevalence compared to significant increases observed in LMICs.^6^ This disparity may be attributed to inadequate funding and structuring of healthcare systems in LMICs, particularly the limited preventive healthcare approach that remains largely oriented towards biological models of disease rather than comprehensive population-level prevention strategies.^1,7^

Historically, substantial progress has been made in global efforts to reduce the risk and incidence of HTN.^8,9^ Over the past three decades, lifestyle modifications, such as the dietary approaches to stop hypertension and increased physical activity, have gained prominence in mitigating HTN risk.^8^ For example, in 1996, a health education initiative in rural Kyowa, Japan, focused on reducing dietary sodium, increasing milk intake, and moderating alcohol and sugar consumption.^9^ Findings from this programme demonstrated effectiveness in reducing systolic pressure levels through non-pharmacological means within the first 6 months and maintaining it for 1.5 years.^9^ With the recognition of biopsychosocial determinants of HTN,^10^ biobehavioural interventions have gained traction in the management of chronic diseases.^11,12^ A biobehavioural intervention refers to an integrated approach that combines biological and behavioural strategies to prevent, manage, or treat disease by targeting both physiological processes (such as autonomic regulation, neuroendocrine responses, or cardiovascular reactivity) and behavioural determinants (such as diet, physical activity, stress management, or treatment adherence.^11,12^ It recognises the reciprocal influence of biological, psychological, and social factors in shaping health outcomes. Several bio-behavioural interventional strategies have emerged, viz., monomodal approaches such as the use of biofeedback for self-regulation of blood pressure,^13^ dietary intervention,^14^ and physical activity interventions.^15^ In addition, multimodal strategies that combine feedback of physiological indices such as peripheral temperature and muscle tension levels, non-biofeedback relaxation techniques (e.g., progressive relaxation, autogenic exercises, meditative procedures), home monitoring of blood pressure, home practice of relaxation, physical exercise, and/or diet^16,17^ were also evident. Notably, multipronged strategies have been more effective in managing essential hypertension than those employing direct blood pressure conditioning.^9,16^

A significant limitation in most trials is the supervised nature or management that involves direct contact between researchers and patients. While researcher- or clinician-administered interventions hold significant value in clinical medicine, non-supervised, education-facilitated, community-wide interventions appear to be more pertinent in public health contexts.^18^ Supervised strategies may fail to empower individuals and communities to assume responsibility for their health, thereby raising concerns about their sustainability.^19^ However, several education-facilitated trials of biobehavioural interventions for HTN have demonstrated promising outcomes.^17,20,21^ For example, in a quasi-experimental study, significant reductions in systolic (−12.4 mmHg) and diastolic (−4.6 mmHg) blood pressures, as well as total cholesterol, were observed following a 3-month educational intervention on lifestyle modification.^20^ Similarly, a recent systematic review provided high-quality evidence supporting education-based, prescribed but unsupervised exercise programmes in reducing blood pressure,^22^ albeit using a monomodal approach.

It remains unclear which educational modes (interactive or noninteractive) are most effective for community-wide interventions. For instance, in a randomised controlled trial (RCT) involving three educational groups, viz., self-learning reading (group 1), monthly regular didactic lectures (group 2), and monthly interactive education workshops (group 3), the findings indicate substantial increases in the proportion of subjects with normalised blood pressure in group 2, with even greater increases in group 3.^23^ Despite the availability of some related systematic reviews, many are limited in scope, focusing on either monomodal or bimodal activities^22,24,25^ or involving education and other supervised activities.^26,27^ Of note, none has investigated education-based psychophysiological biobehavioural strategies, thus warranting the review of all existing community-based, education-based biobehavioural interventions to identify effective components and their mechanisms. This study is thus essential for designing future community-wide, education-based biobehavioural intervention strategies for the prevention and control of hypertension.

### Review objective

To systematically evaluate and synthesise evidence on the effectiveness of multi-pronged community-based biobehavioural education interventions in reducing blood pressure among adults with or at risk of hypertension.

## Methods

### Protocol and registration

This is a systematic review focusing on community-based control strategies for hypertension. The review adhered to the guidelines outlined in the Preferred Reporting Items for Systematic Reviews and Meta-Analysis checklists (Supplemental Material 1). The review protocol is registered with the International Prospective Register of Systematic Reviews (PROSPERO; ID: CRD420251034159). There is an amendment to the protocol post-registration. Mr. Innocent Nweke did not participate in the review as initially proposed. One other reviewer, Bernard Appiah was co-opted during the review process.

### Eligibility

Studies were included if they met the following inclusion criteria:

*Population*: Studies focusing on individuals with or without HTN. Participants of any age, sex or socio-economic status
*Intervention*: Multipronged biobehavioural interventions, which may include any combination of three or more feedback of physiological indices, viz., peripheral temperature and muscle tension levels, progressive relaxation, autogenic exercises, mediative procedures, home monitoring of BP, home practice of relaxation, physical exercise, diet, smoking cessation, alcohol cessation, consumption of green vegetable, reduced salt intake, etc.
*Comparison*: Randomised community trials were preferred; however, other non-RCTs were included.
*Outcomes*: The outcomes are the mean systolic and diastolic blood pressure.
*Timeframe*: Database was searched from the inception of each database up until May 2025.
*Language*: All studies were included irrespective of language. Articles published in languages other than English were translated with the aid of Google Translate.
Studies were excluded based on the following exclusion criteria:
*Population studies*: All studies involving participants with cardiovascular disease and risk factors, without a distinct statistical analysis for HTN.
*Intervention studies*: All efficacy studies that examined the efficacy of pharmacological agents or a combination of pharmacological agents and lifestyle modification, without a distinct analysis for lifestyle intervention.
*Comparison studies*: Observational designs may be employed in community trials; however, studies with no clear control cluster were excluded.
*Outcomes*: Studies that did not report the primary endpoint, namely, mean difference in blood pressure, gestational hypertension, hypertension risk or incidence, will be excluded. Similarly, implementation studies that did not report at least an implementation outcome were excluded.

### Sources of information

Searches were conducted across multiple databases, namely PubMed, MEDLINE, the Cumulative Index of Nursing and Allied Health Literature (CINAHL), the Cochrane Library, Academic Search Complete, Scopus, Web of Science, and African Journals (SABINET). These searches covered the period from the databases’ inception until May 2025. In cases where access to the full texts of qualifying articles was limited or inaccessible, the study authors were contacted by the research team.

### Search strategy

The primary reviewer, together with an information specialist with significant expertise in literature searches, designed the search strategy. The trial search strategy was initiated by extracting search terms from five pivotal articles^14,16,17,20,23,27^ and subsequently determining their synonyms, referred to as “exact terms,” using the Cochrane Library’s “Medical Terms” feature. Ultimately, a combination of MeSH terms and free search terms was selected and trialled using PubMed. Sensitivity was assessed at face value, using the number of eligible articles in the first two pages of the research results. Based on this strategy, an initial PubMed search yielded 3340 studies. The tested search strategy was adapted to align with the syntax and subject headings of the other databases, including MEDLINE, the Cochrane Library, Academic Search Complete, CINAHL, Scopus, Web of Science, and African Journals (SABINET). The PubMed strategy is provided thus:

(((High blood pressure OR hypertension or blood pressure[MeSH Terms]) AND (Lifestyle OR behavior OR exercise OR physical activity OR diet OR supplement, food OR supplementations, dietary OR potassium OR salt intake OR Salt OR education OR food labelling OR food Labeling OR weight loss OR weight loss diet OR weight loss agents OR running OR screening OR dancing OR fruits or vegetable OR smoke OR alcohol or air pollutants or green spaces OR biobehavioral OR Behavior Therapy / methods OR Biofeedback, Psychology OR Combined Modality Therapy OR Muscle Relaxation OR Hot Temperature OR Baths OR Hydrotherapy OR Biofeedback[MeSH Terms])) AND (education OR health education OR health education, community OR patient education[MeSH Terms]) AND (Cluster randomisation OR Cluster* or community trial or community-wide[MeSH Terms])

### Data management and study selection

After conducting the literature review and identifying the data from various databases, the references were imported into EndNote 21 for streamlined management. The first step involved removing duplicate entries, followed by a thorough screening of the remaining articles based on their titles and abstracts. This process was conducted by two independent reviewers (MN) and Innocent Nweke. Conflicts were resolved through discussion, and the final selection of articles adhered to predetermined eligibility criteria. This was followed by obtaining the full texts of articles that had passed the initial screenings. If full texts were unavailable, the primary reviewer contacted the study authors via email. A lack of response served as an additional exclusion criterion, and the full texts of the chosen articles were subsequently downloaded using EndNote 21.

### Data collection

Data were extracted by a trained research assistant using a pre-specified template and independently verified by the primary reviewer (MN) against the original articles. Any discrepancies were resolved through discussion and review of the full text until agreement was reached.

### Outcomes and data items

The primary outcomes were changes in systolic blood pressure (SBP) and diastolic blood pressure (DBP) following community-based biobehavioural educational interventions. Mean differences (MD) between intervention and control groups at follow-up were extracted. For pre–post studies without control groups, post-intervention measurements were treated as the intervention condition and pre-intervention values as the comparator. Data extracted included study characteristics (author, year, country, study design), participant sample sizes, intervention components, duration, and follow-up period. Numerical data collected for meta-analysis comprised mean blood pressure values, measures of variability, and the number of participants analysed in each arm. These variables also supported subgroup and sensitivity analyses.

### Risk of bias assessment

The potential bias in the eligible individual studies was evaluated using the Cochrane risk of bias tool (RoB-2) and Risk of Bias In Non-randomised Studies of Interventions I (ROBINS-I), which includes five domains. The rationale for choosing this tool is linked to community trials being implemented using randomised^28^ or non-randomised design.^29^

#### Effect measures

The effect measure used was the MD in SBP and DBP between intervention and comparator groups at follow-up. Mean values and measures of variability were extracted to compute comparable estimates. Where necessary, standard errors were derived from reported statistics. For pre–post studies without a control group, post-intervention values were treated as the intervention condition and pre-intervention values as the comparator. All effects were expressed in mmHg.

### Data synthesis

We summarised sociodemographic and study characteristics using frequencies, means, and standard deviations. A narrative synthesis was employed to describe the results of individual studies. Meta-analyses were conducted using a random-effects inverse-variance model to account for expected variability across studies. Analyses were performed in R (R Foundation for Statistical Computing, Vienna, Austria) using the meta and metafor packages. Between-study heterogeneity was quantified using the *I*^2^ statistic and Cochran’s *Q* test. Heterogeneity exceeding 50% was considered substantial.^30^ Prediction intervals were calculated to assess the expected range of intervention effects across settings. Prespecified subgroup and sensitivity analyses were conducted to explore potential sources of heterogeneity.

### Additional analyses

#### Meta-bias

The funnel plot was visually examined to detect publication bias, and Egger’s test was used to statistically evaluate the symmetry of the funnel plot. A non-significant test result suggests symmetry in the funnel plot, indicating no publication bias. Meta-regression was applied to investigate the impact of moderator variables such as study design, age, gender, and others.

#### Sensitivity

To assess how potentially questionable decisions during the review process might affect the meta-analysis outcomes, a sensitivity analysis was conducted using the “one study removed” technique.^31^ This method identified studies as outliers if their effect size (%) fell outside the 95% confidence interval of the average effect size.

### Grading the strength of evidence

#### Procedure

To assess the certainty of the synthesised evidence, the Grading of Recommendations, Assessment, Development, and Evaluation (GRADE) framework was employed.^32^ The certainty of evidence was evaluated across five key domains: risk of bias, inconsistency, indirectness, imprecision, and publication bias.^32,33-37^ This assessment offered a structured approach for determining the confidence level in the collective evidence that underpins the study conclusions. By utilising systematic grading, this review aimed to ensure that recommendations are grounded in evidence-based insights, thereby enhancing HTN stratification strategies.

#### Interpretation of evidence

According to the GRADE framework,^32,33^ the overall quality of evidence was evaluated as follows: High certainty indicated that further research is unlikely to alter confidence in the effect estimate. Moderate certainty suggested that additional studies may influence confidence in the effect estimate. Low certainty implied that further research is expected to impact confidence in the estimates. Very low certainty denotes that the actual effect is highly uncertain due to methodological limitations. The criteria for downgrading included a high or unclear risk of bias in 50% or more of the included studies, resulting in a one-level downgrade of the evidence. The final grading was maintained at its original level if the studies demonstrated a low or moderate risk of bias.^32^

## Results

### Study selection

**Figure 1.**
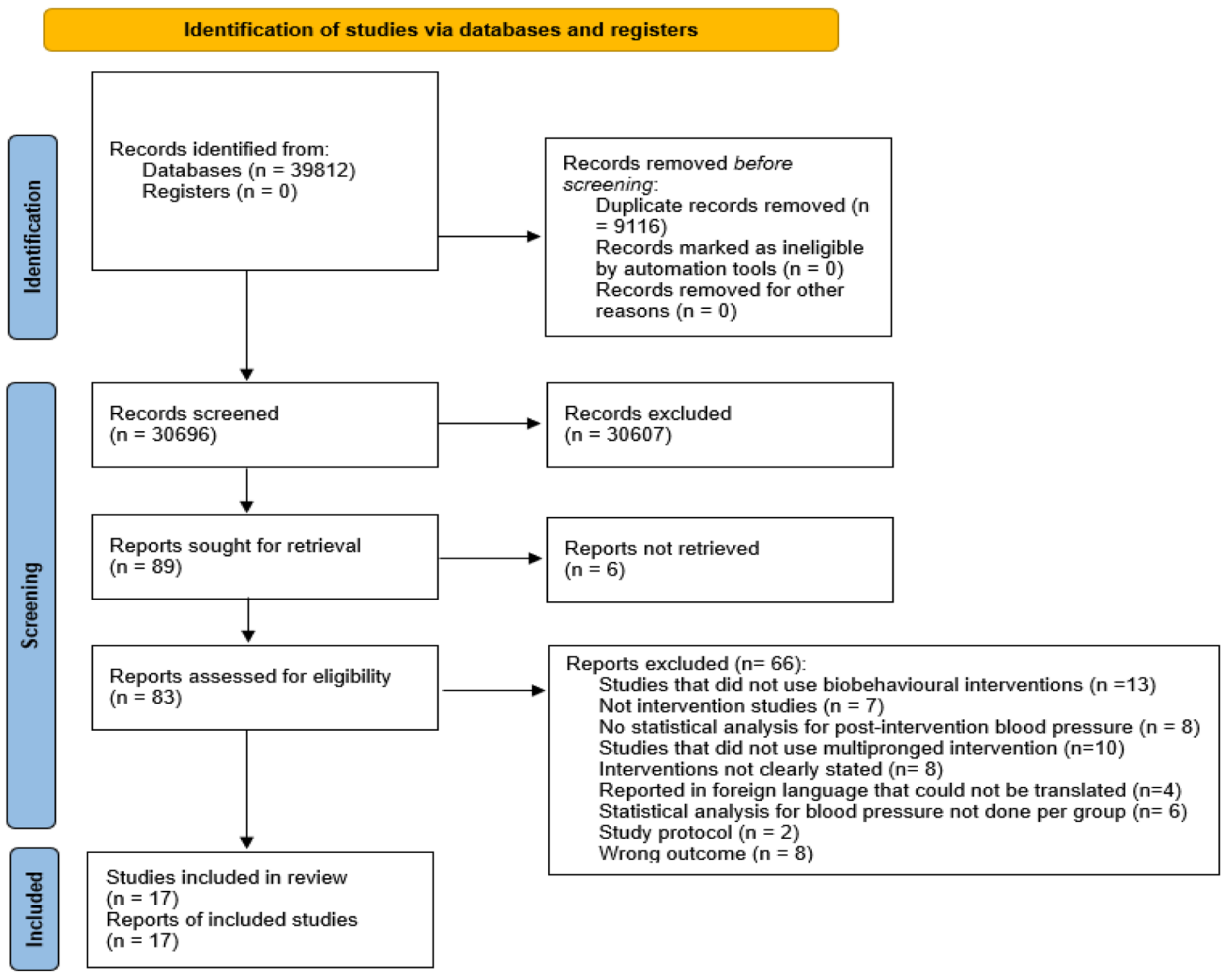
PRISMA flow diagram of study selection process for the systematic review.

Following the literature search, we retrieved 39,812, out of which 9116 were duplicates. Following the elimination of duplicates, 30,646 articles remained for initial title and abstract screening. Following this screening, 30,557 articles were ineligible and excluded. A total of 89 full-text studies were subsequently subjected to further data screening, and data were extracted from 17 eligible articles (Figure 1).

### Study characteristics

A total of 5221 unique participants were included across the 17 eligible studies. For the meta-analysis, arm-level sample sizes were used, yielding 3683 participants in the intervention groups and 3754 in the control or usual-care groups. For studies with pre–post designs, post-intervention measurements were treated as the intervention arm and pre-intervention measurements as the control condition. Of 17 studies, 4 (*n* = 1304) were from the United States,^38–41^ 4 (*n* = 538) from Iran,^42–45^ 2 (*n* = 386) from Italy^46,47^ and 1 each from Thailand,^48^ Egypt,^49^ Philippines,^50^ India,^51^ Uganda,^52^ China,^53^ and Nepal,^54^ respectively. Fourteen studies were RCTs,^38–48,50,53,54^ and three were non-RCTs.^49,51,52^ In 12 studies, the hypertension type was not specified.^39,41,43–45,47–51,53^ Of the remaining five studies, hypertension was classified as arterial hypertension,^45^ primary hypertension,^41,52^ mild hypertension,^38^ and uncontrolled hypertension.^39^ Sample size ranged from 60, as reported in Jafari et al.,^43^ to 2016 by Kwiringira et al.^52^ The mean age of participants was 57.98 ± 9.47 years. Most studies (76.47%) had a greater percentage of the female population (Supplemental Material 2).

### Risk of bias assessment

**Figure 2.**
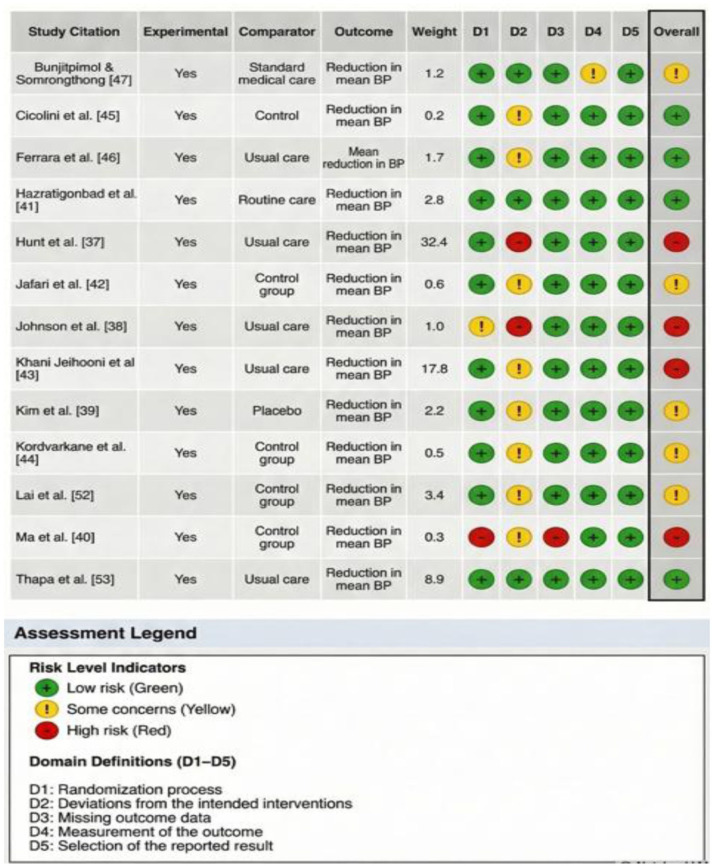
Risk of bias assessment for RCT. RCT: randomised controlled trial.

Across the non-randomised studies, two showed moderate risk of bias,^51,52^ while two demonstrated serious concerns due to lack of control groups and confounding bias.^49,51^ Collectively, these studies offer useful implementation insight but limited causal certainty (Supplemental Material 3). Among the randomised trials, the most suitable methodologies were applied in the majority of the studies, with the strongest methodological rigour/quality observed in studies reported by HazratiGonbad et al.,^42^ Lai et al.,^53^ and Thapa et al.^54^ In contrast, a higher risk was noted in studies reported by Hunt et al.,^38^ Johnson et al.,^39^ and Ma et al.,^41^ due to deviations from intended interventions and missing data. Overall, the trial evidence reflects moderate risk of bias, with more recent studies generally demonstrating improved methodological rigour (Figure 2).

### Results of individual studies

A summary of findings in terms of the risk estimate and corresponding confidence interval, and/or *p*-value was reported. To aid interpretation, the summary of findings, the design, setting, and effect size type are reported (Supplemental Material 4).

### Mode of delivery

The following health professionals were involved in the intervention administration: nurses,^40,43–46,50^ community health workers,^41,52,54^ physicians,^38–40,45,47,50,51^ and Dieticians or nutritionists.^46,47,50^ More than half of the studies^39–41,43,47–52^ utilised face-to-face delivery with a peer-support network.

### Narrative synthesis

Across all the included studies, lifestyle modification interventions targeting diet, physical activity, smoking, alcohol intake, stress management, and weight control demonstrated varying effects on blood pressure reduction. The interventions were delivered as multi-pronged programmes combining dietary counselling, exercise promotion, and behavioural support, with durations ranging from 1 month to 5 years.

#### Effects on SBP

Across the 17 included studies, the direction and statistical significance of SBP changes varied, although most studies favoured the intervention. Several studies reported statistically significant reductions in SBP among participants receiving the intervention compared with control groups, including those conducted by Hunt et al.,^38^ Khani Jeihooni et al.,^44^ Ferrara et al.,^47^ Gabiola et al.,^50^ Kordvarkane et al.,^45^ Kwiringira et al.,^52^ Lai et al.,^53^ and Thapa et al.^54^ These trials implemented multi-component educational or behavioural interventions and demonstrated lower SBP values among intervention participants relative to controls. Other RCTs reported non-significant reductions in SBP, although the direction of effect generally favoured the intervention. These included studies by Bunjitpimol et al.,^48^ Cicolini et al.,^46^ HazratiGonbad et al.,^42^ Jafari et al.,^43^ Johnson et al.,^39^ Kim et al.,^40^ and Ma et al.^41^ In these studies, intervention participants typically exhibited modest decreases in SBP or slightly lower SBP levels than controls, but the confidence intervals crossed the null value. One study reported higher SBP in the intervention group relative to controls, as observed in the trial by James et al.,^51^ although this difference was not statistically significant. Two non-randomised studies using pre–post designs also suggested improvements following the intervention. Elgendy et al.^49^ reported a reduction in mean SBP from 141.7 mmHg at baseline to 136.8 mmHg after the intervention, while Kwiringira et al.^52^ observed a reduction from 159 to 149 mmHg during the intervention period (Supplemental Material 4). Overall, while the magnitude and statistical significance of effects varied across studies, the majority of trials reported reductions in SBP associated with the interventions, consistent with the direction of the pooled meta-analytic estimate, although substantial heterogeneity was observed across studies.

#### Effects on DBP

Six RCTs reported significant reductions in DBP on implementing comprehensive multi-modal educational interventions.^41,43–46,50^ Interestingly, there was no disparity in the direction of effect, as intervention participants showed lower DBP compared to controls in all the studies. In addition, six RCTs^38,40,42,47,48,54^ and one non-randomised controlled trial (NRCT)^51^ reported non-significant reductions in DBP on implementing comprehensive multi-modal educational interventions. Remarkably, intervention participants showed lower DBP compared to controls in all the studies.^39-46,50,51,53,54^ An exception is Hunt et al.,^38^ where no significant difference was noted between the DBP of the intervention and control groups, and in James et al.^51^ and Thapa et al.,^54^ where the DBP of the intervention participants were higher than that of the controls. Once again, in the NRCTs without a control group, improvements were again noted in pre–post analyses. Elgendy et al.^49^ reported a reduction from 91.6 to 87.2 mmHg, while Kwiringira et al.^52^ documented a decrease from 97 to 92 mmHg (Supplemental Material 4).

### Meta-analysis

#### Efficacy of education-based biobehavioural intervention (BBI) for SBP control

**Figure 3.**
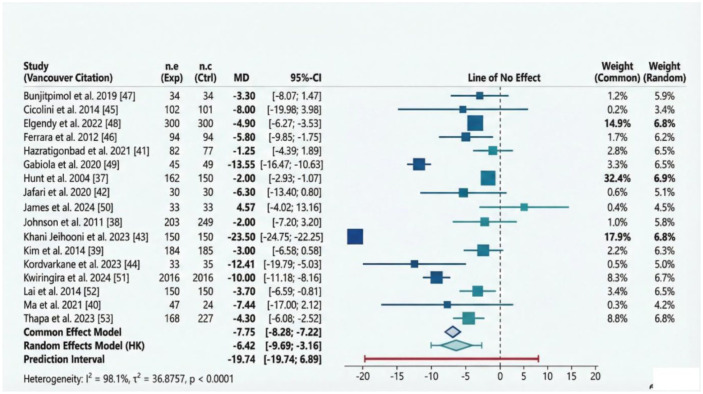
Forest plot displaying the efficacy of education-based biobehavioural interventions for blood pressure control (systolic blood pressure).

There was a statistically significant and positive improvement in SBP among participants following education-based bio-behavioural interventions (MD: −6.42 mmHg, 95% CI: −9.69 to −3.16). The prediction interval is wide (MD: −19.74 to 6.89 mmHg), indicating that effects vary across settings. We observed a substantial degree of heterogeneity (*I*^2^ = 98.1% Figure 3).

##### Sub-group analysis

This is based on intervention category, study design and study duration.

**Figure 4.**
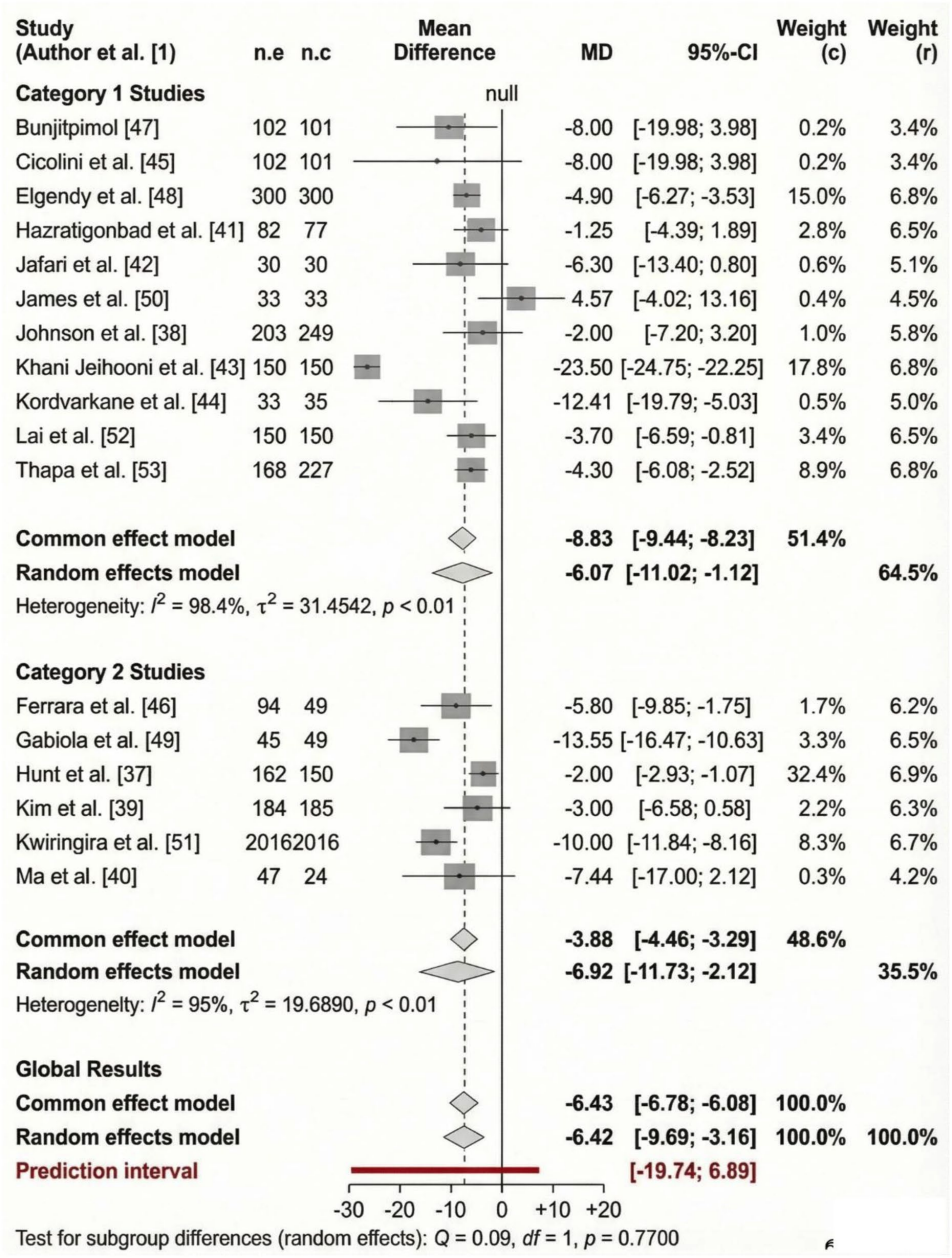
Forest plot sub-group analysis displaying the efficacy of intervention categories 1 (⩾4 components) and 2 (2–3 components) for systolic hypertension.

*Intervention category.* When the analysis was limited to intervention category 1, a statistically significant positive effect (reduction in SBP) was observed (MD: −6.07, CI: −11.02 to −1.12); however, the heterogeneity remained substantial (*I*^2^ = 98.4%; Figure 4). When the analysis was limited to intervention category 2, a statistically significant positive effect was observed (MD: −6.92, CI: −11.73 to −2.12). The heterogeneity remained substantial (*I*^2^ = 95%; Figure 4).

**Figure 5.**
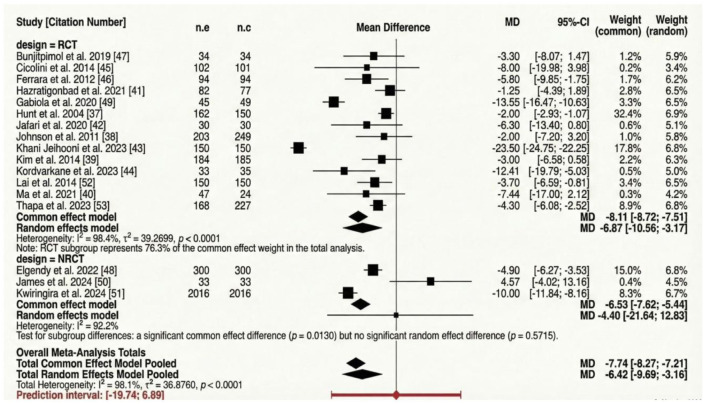
Forest plot subgroup analysis displaying the efficacy of Intervention study designs for systolic hypertension.

*Study design.* Limiting the analysis to RCT design, a statistically significant positive effect was observed (MD: −6.87, CI: −10.56 to −3.17). The degree of heterogeneity remained substantial (*I*^2^ = 98.4%; Figure 5). When the analysis was limited to NRCT, there was no statistically significant difference observed (MD: −4.40, CI: −21.64 to 12.83). However, the degree of heterogeneity remained substantial (*I*^2^ = 92.2%; Figure 5).

**Figure 6.**
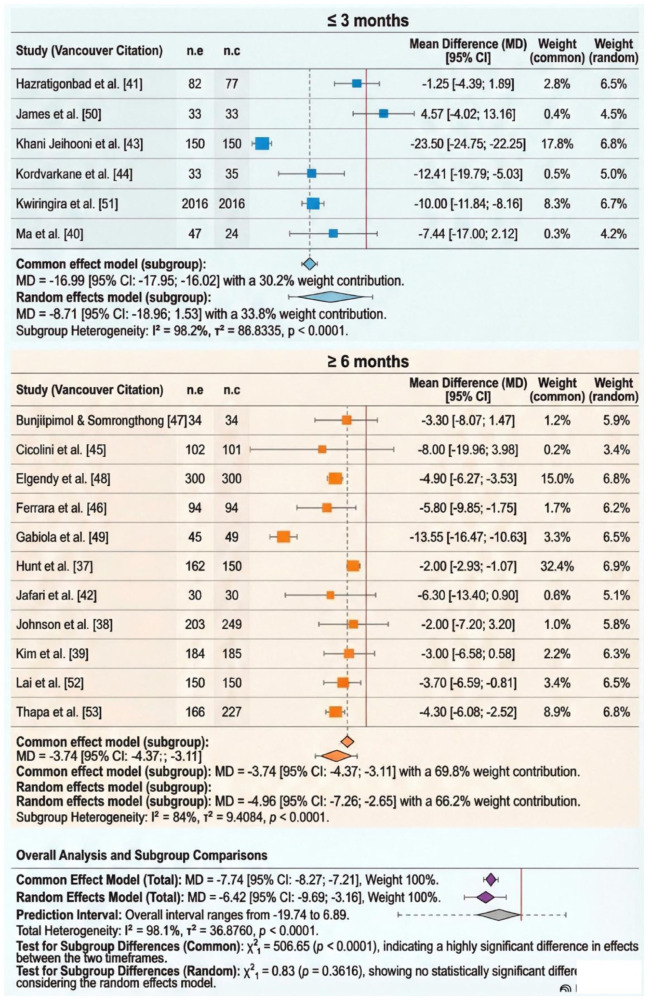
Forest plot subgroup analysis displaying the efficacy of intervention (study duration) for systolic hypertension.

*Intervention duration.* When the analysis was limited to intervention ⩽3 months, no statistically significant difference was observed (MD: −8.71, CI: −18.96 to 1.53). The heterogeneity remained substantial (*I*^2^ = 98.2%; Figure 6). When the analysis was limited to intervention ⩾6 months, a statistically significant difference was observed (MD: −4.96, CI: −7.26 to −2.65). The heterogeneity remained substantial (*I*^2^ = 84%; Figure 6).

##### Sensitivity analysis

**Figure 7.**
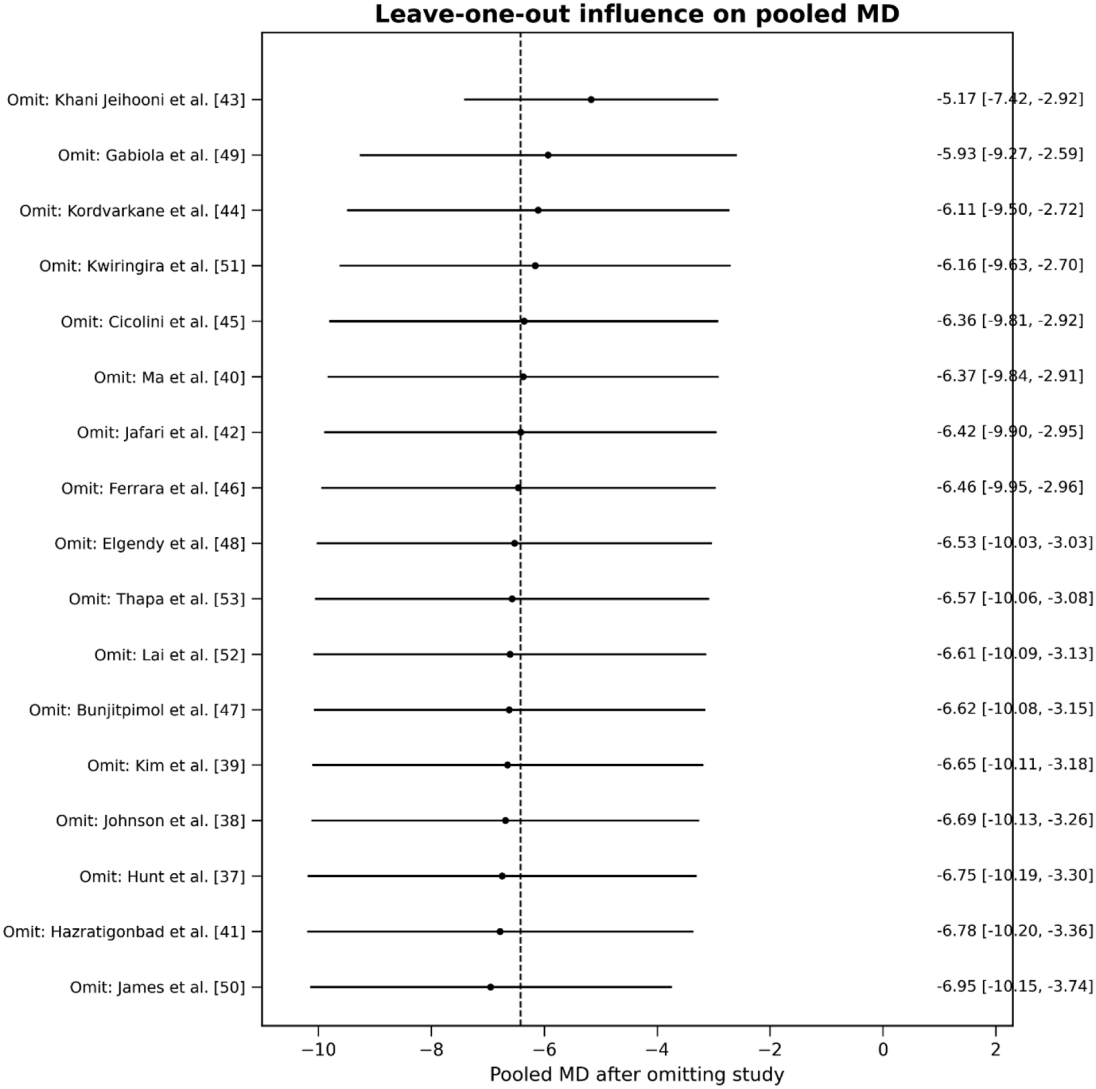
Forest plot showing the influence of the leave-one-out analyses on pooled MD. MD: mean difference.

The omission of Khani Jeihooni et al.^44^ and Gabiola et al.^50^ causes moderate shifts in the pooled MD (around −5.17 to −5.93 mmHg). However, omitting other studies^38-43,45–49,51–54^ resulted in a pooled MD of around −6.11 to −6.95 mmHg, which is very close to the overall estimate, indicating minimal shifts. Overall, no single study dictates the direction of the pooled results. This supports the stability of the overall findings, despite the very high heterogeneity noted earlier. The blood pressure-lowering effect remains consistent across leave-one-out analyses (Figure 7).

##### Meta-bias

*Main analysis.* Regarding the efficacy of education-based BBI for SBP control, the funnel plot does not show clear directional asymmetry. Egger’s linear regression test was non-significant, *t* = 0.28, *df* = 15, *p* = 0.7811, with a bias (intercept) estimate of 0.8603, SE = 3.0401 (see Supplemental Figure 8 in Supplemental Material 5).

*Sub-group.* The meta-bias for subgroup analyses based on intervention category, study design and study duration are outlined below:

*Intervention category.* For intervention category 1 (⩾4 components), the funnel plot does not show clear directional asymmetry. Egger’s linear regression test was non-significant, *t* = 1.21, *p* = 0.2572, with a bias (intercept) estimate of 4.7068, SE = 4.8431 (see Supplemental Figure 9 in Supplemental Material 5). For intervention category 2, the funnel plot does not show clear directional asymmetry (see Supplemental Figure 10 in Supplemental Material 5). However, Egger’s linear regression test was non-significant, *p* = 0.2785, which indicates no publication bias.

*Study design.* For the RCTs, the Funnel plot indicates asymmetry, with the points distributed across both sides of the pooled effect estimate (see Supplemental Figure 11 in Supplemental Material 5). However, Egger’s linear regression test was non-significant, *t* = 0.30, *p* = 0.7711, with a bias (intercept) estimate of 1.1111, SE = 4.2466. This indicates no publication bias. For NRCT, the visual impression of asymmetry (points scattered unevenly) may simply reflect chance due to the small size of *k*. The Egger’s test was not computed as *k* (number of studies) was less than three (see Supplemental Figure 12 in Supplemental Material 5).

*Intervention duration.* For study duration ⩽3 months, the visual impression of asymmetry (points scattered unevenly) may simply reflect chance due to the small size of *k* (see Supplemental Figure 13 in Supplemental Material 5). The Egger’s test was not computed as *k* (number of studies) was less than three. For study duration ⩾6 months, the funnel plot does not show clear directional asymmetry. Egger’s linear regression test was non-significant, *t* = −1.33, *p* = 0.2152, with a bias (intercept) estimate of −1.6314, SE = 1.3122 (see Supplemental Figure 14 in Supplemental Material 5).

#### Efficacy of education-based BBI for DBP control

The analyses across 17 studies with 6134 participants in the intervention arms and 6451 in the control, the random-effects MD in DBP favours the intervention by −3.04 mmHg, 95% CI −6.02 to −0.07. The prediction interval is wide, −15.89 to 9.80 mmHg, which crosses the null and signals that effects vary across settings. Heterogeneity is very high: *I*^2^ = 99.3%, τ^2^ = 35.0007, *p* = 0 (see Supplemental Figure 15 in Supplemental Material 5).

##### Sub-group analysis

This was based on study design (RCT and non-RCT) and intervention (viz., categories 1 and 2), duration of intervention (⩽3 and ⩾6 months). Category 1 interventions included four or more components (e.g., exercise, diet, stress management, heat therapy, alcohol, smoking, obesity, relaxation). Category 2 comprised two or three components, viz., exercise, diet, stress management, heat therapy, alcohol, smoking, obesity, and relaxation.

*Study design.* Limiting the analysis to RCT design, a statistically significant effect was observed (MD: −3.74, CI: −7.25 to −0.22); however, the heterogeneity remained substantial (*I*^2^ = 99.2%). Limiting the analysis to the NRCT design, a statistically non-significant difference was observed (MD: 0.03, CI: −0.60 to 0.66). However, the heterogeneity decreased (*I*^2^ = 0%; see Supplemental Figure 16 in Supplemental Material 5).

*Intervention category.* When the analysis was limited to intervention category 1, a statistically non-significant effect was observed (MD: −3.31, CI: −8.33 to 1.71), yet the heterogeneity remained substantial (*I*^2^ = 99.4%). Similarly, for intervention category 2, a statistically non-significant effect was observed (MD: −2.55, CI: −5.65 to 0.54), and the heterogeneity remained substantial (*I*^2^ = 92.7%; see Supplemental Figure 17 in Supplemental Material 5).

*Study duration.* When the analysis was limited to intervention ⩽3 months, no statistically significant difference was observed (MD: −5.85, CI: −13.73 to 2.04); however, the heterogeneity remained substantial (*I*^2^ = 99.6%). Likewise, when the analysis was limited to intervention ⩾6 months, no statistically significant difference was observed (MD: −1.40, CI: −3.32 to 0.52). The heterogeneity, however, remained substantial (*I*^2^ = 89.1%; see Supplemental Figure 18 in Supplemental Material 5).

##### Sensitivity analysis

When Khani Jeihooni et al.^44^ were omitted, the pooled MD rose to −1.77 mmHg (95% CI −3.37 to −0.18), indicating a large shift. This is the least negative estimate and indicates that this study had a strong influence in pulling the overall effect size downward. Moderate shifts were also observed through the omission of Kordvarkane et al.,^45^ Gabiola et al.,^50^ Ma et al.,^41^ Cicolini et al.,^46^ and Jafari et al.,^43^ which moved the pooled MD closer to zero (around −2.7 to −2.9 mmHg). Omitting additional studies^38–40,42,47–49,51–54^ leaves the pooled MD around −3.1 to −3.4 mmHg, very close to the overall estimate, also indicating minimal shifts. Overall, no single study dictates the direction of the pooled result. Of note, Khani Jeihooni et al.^44^ appear to be the most influential, but even without it, the pooled effect is still statistically significant and negative. This supports the stability of the overall findings, despite the very high heterogeneity noted earlier. The blood pressure–lowering effect remains consistent across leave-one-out analyses, though its magnitude is somewhat sensitive to large-effect studies, as observed by Khani Jeihooni et al.^44^ (see Supplemental Figure 19 in Supplemental Material 5).

##### Meta-bias

Regarding the efficacy of education-based multi-pronged biobehavioural intervention (MBBI) for DBP, the funnel plot does not show clear directional asymmetry. Egger’s linear regression test was non-significant, *p* = 0.0573 (see Supplemental Figure 20 in Supplemental Material 5). The meta-bias for subgroup analyses based on intervention category, study design and study duration are outlined below:

*Study design.* For the RCT design, the funnel plot indicates asymmetry, with the points not evenly spread around the pooled estimate (see Supplemental Figure 21 in Supplemental Material 5). Also, Egger’s linear regression test was significant for RCT designs (*p* < 0.001), indicative of publication bias. However, when limited to NRCT design, the funnel plot does not show clear directional asymmetry, and Egger’s linear regression test was non-significant (*p* = 0.15830), indicating no publication bias (see Supplemental Figure 22 in Supplemental Material 5).

*Intervention category.* For Intervention category 1 (⩾4 components), the points are not evenly spread around the pooled estimate. Most studies are clustered on the right (X), with fewer on the left. The Egger’s linear regression test was also significant for intervention category 1 (*p* = 0.0332), indicative of publication bias (see Supplemental Figure 23 in Supplemental Material 5). For Intervention category 2 (two-three components), the funnel plot revealed no clear directional asymmetry, but the Egger’s linear regression test was non-significant (*p* = 0.5435), suggestive of no publication bias (see Supplemental Figure 24 in Supplemental Material 5).

*Duration of intervention.* For ⩽3 months of study duration, the points are not evenly spread around the pooled estimate. Most studies are clustered on the left, with fewer on the right (see Supplemental Figure 25 in Supplemental Material 5). However, Egger’s linear regression test was non-significant for intervention ⩽3 months duration (*p* = 0.4716), indicative of no publication bias. For ⩾6 months study duration, the funnel plot indicates asymmetry, with the points distributed across both sides of the pooled effect estimate see Supplemental Figure 26 in Supplemental Material 5). Likewise, Egger’s linear regression test was non-significant for intervention ⩾6 months duration (*p* = 0.1964), indicative of no publication bias.

#### Certainty of evidence

Evidence shows clinically meaningful reductions in SBP and DBP with MBBIs. Certainty for SBP is moderate due to consistent direction of effect and reasonable precision. Certainty for DBP is low–moderate owing to greater dispersion and borderline precision across studies (Supplemental Material 6).

## Discussion

This systematic review and meta-analysis synthesised data from 17 studies involving 5532 hypertensive adults to determine the effect of education-based MBBIs on blood pressure control. The pooled findings showed significant reductions in both SBP and DBP, confirming that structured behavioural and educational programmes meaningfully contribute to hypertension management. These results reinforce a growing evidence base that emphasises patient education, self-management, and lifestyle modification as integral components of cardiovascular risk reduction.

### Overall interpretation

The pooled MD of −6.42 mmHg for SBP and −3.04 mmHg for DBP suggest that education-based MBBIs may contribute to improved blood pressure control among adults with hypertension. These reductions are clinically meaningful, as prior evidence indicates that even modest decreases in SBP are associated with substantial reductions in major cardiovascular events, including stroke and myocardial infarction.^55^ Similar magnitudes of blood-pressure reduction have been reported in lifestyle-based and behavioural intervention trials such as the PREMIER study and other multidisciplinary education programmes.^56-60^ However, interpretation of the pooled estimates should be undertaken cautiously. Both SBP and DBP analyses demonstrated very high heterogeneity (*I*^2^ > 90%), reflecting substantial variability in intervention characteristics, populations, and study contexts. Furthermore, the prediction intervals were wide, and for DBP the interval crossed the null value, indicating that the magnitude—and in some cases the direction—of the intervention effect may vary across settings. This variability likely reflects differences in programme intensity, behavioural components, follow-up duration, and healthcare delivery contexts across the included studies.^61,62^ Accordingly, the pooled estimates should be interpreted as an average effect across heterogeneous interventions rather than a universally generalisable treatment effect.

### Systolic blood pressure

More than half of the included studies (52.9%) reported significant reductions in SBP, and the pooled estimate (−6.42 mmHg; 95% CI −9.69 to −3.16) aligns with previous meta-analyses on lifestyle education interventions.^61,62^ Both multi-pronged (⩾4 components) and moderately intensive (two–three elements) interventions produced comparable effects, indicating that consistent engagement and quality of delivery may be more critical to success than the number of targeted behaviours.^63^ RCTs produced stronger reductions (−6.87 mmHg) than non-randomised studies (−4.40 mmHg), reflecting the improved internal validity of randomised designs. Intervention duration also influenced effectiveness: programmes extending beyond 6 months achieved more durable improvements.^64,65^ These findings support behavioural theory, suggesting that maintenance of new habits and self-efficacy requires reinforcement and social support over time.

### Diastolic blood pressure

For DBP, the pooled MD of −3.04 mmHg (95% CI −6.02 to −0.07) confirms the benefit of MBBIs, which corroborates the meta-analysis reported by Foroumandi et al.,^66^ which highlights a −3.87 mmHg decrease in DBP following structured education. Although smaller in magnitude than SBP reductions, these variations remain clinically useful, as even a 2 mmHg decline in DBP reduces coronary heart disease risk by about 6% and stroke mortality by 15%, as previously documented.^67^ Once more, RCTs revealed stronger effects (−3.74 mmHg) than non-RCTs, suggesting that structured implementation and monitoring enhance efficacy. Interventions addressing multiple lifestyle factors tended to yield larger reductions, though subgroup differences were not statistically significant, possibly reflecting variations in adherence, intensity, and cultural context.^68^ Longer interventions produced steadier DBP reductions, emphasising the time-dependent nature of behavioural adaptation.^69^ The strong effect of the programme by Khani Jeihooni et al.^70^ underscores how theory-based education frameworks grounded in constructs such as self-efficacy, perceived benefits, and behavioural intention can amplify impact.

### Possible mechanisms of effect

Our findings demonstrate that MBBIs reduce blood pressure through complementary behavioural and physiological pathways. In addition, education enhances awareness and health literacy, leading to improved dietary choices, medication adherence, and stress regulation. Behavioural counselling and peer reinforcement, together with psychological mechanisms, further promote physical activity, weight loss, and reduction of alcohol and sodium intake, which collectively improve vascular elasticity and autonomic balance. Moreover, mindfulness-based and relaxation techniques attenuate sympathetic overactivity and cortisol release, resulting in lower resting blood pressure. Neurophysiological evidence shows that such approaches modulate vagal tone and inflammatory cytokine profiles, leading to systemic cardiovascular benefits.^71,72^ In digital and hybrid formats, continuous feedback, goal tracking, and social accountability further strengthen behavioural reinforcement and long-term adherence.^73,74^

### Heterogeneity and publication bias

The very high heterogeneity observed in both SBP and DBP analyses likely reflects differences in intervention components, delivery methods, duration, and population characteristics across studies. The included trials were conducted across multiple regions, including Asia, Africa, Europe, and North America, and varied in their implementation approaches, ranging from community health worker–led education to nurse-led counselling and digital or telephone-based follow-up. Such variation can influence behavioural adherence, intervention intensity, and baseline cardiovascular risk, thereby contributing to differences in observed effects. Although funnel plot inspection and Egger’s regression suggested limited evidence of publication bias overall, the wide prediction intervals indicate that intervention effectiveness may vary considerably across contexts.

### Relation to previous evidence and guidelines

These findings are congruent with recent systematic reviews that underscore the value of patient education and behavioural change in hypertension control.^75,76^ A 2024 meta-analysis of telehealth-based self-management interventions reported pooled SBP and DBP reductions of 5.7 and 1.9 mmHg, respectively.^77^ Similarly, community-based interventions in sub-Saharan Africa showed mean SBP reductions of 6–8 mmHg when education was integrated into routine care.^78^ The review outcome affirms the current global frameworks, such as the WHO HEARTS technical package and the World Hypertension League’s “Call to Action,” both of which emphasise education, self-monitoring, and team-based care as cornerstones of population-level hypertension management.^79^ These interventions are consistent with Sustainable Development Goal 3.4, which aims to reduce premature mortality from non-communicable diseases by one-third by 2030.

### Strengths and limitations

Broad geographical representation, the inclusion of diverse study designs, and the use of detailed subgroup and sensitivity analyses strengthen the interpretability of the findings. The generally consistent direction of effects across studies suggests reasonable external validity across varied cultural and socioeconomic settings. However, several limitations should be acknowledged. The very high heterogeneity observed across studies reduces the precision and generalisability of the pooled estimates. In addition, most studies did not implement blinding, which may introduce performance bias. Evidence of small-study effects was also detected for the DBP outcome within the RCT subgroup (Egger’s test *p* < 0.001), indicating possible publication bias and warranting cautious interpretation of the pooled DBP estimate. Adherence and intervention fidelity were inconsistently assessed and were frequently based on self-report, and follow-up periods were relatively short in many trials, limiting insight into long-term sustainability. Moreover, the type of hypertension was not specified in the majority of included studies (12 of 17), making it difficult to distinguish between essential, secondary, or gestational hypertension and potentially contributing to outcome heterogeneity. Finally, only a small number of studies were conducted in low-income countries, leaving important evidence gaps for the most resource-constrained settings.

### Implications for health systems and research

The beneficial effects of MBBIs documented in this study highlight their potential as scalable, low-cost adjuncts to pharmacological therapy. Integrating structured education into routine hypertension management could strengthen control rates at the population level, particularly in primary-care and community settings where resources are limited. Task-sharing models involving nurses, physiotherapists, and community health workers could facilitate programme delivery while maintaining cost efficiency. Digital delivery platforms and peer-support networks can further extend reach, providing continuous reinforcement and progress monitoring. Future trials should prioritise standardised intervention frameworks, explicit reporting of adherence measures, and cost-effectiveness analyses. Longitudinal research examining sustainability beyond 12 months and real-world implementation fidelity will be essential to guide policy and scale-up efforts.

## Conclusion

Education-based MBBIs are associated with modest improvements in SBP and DBP among adults with hypertension. However, the very high heterogeneity and wide prediction intervals observed in this review suggest that intervention effects vary across populations, settings, and programme designs. These findings indicate that such interventions may be beneficial overall but should be interpreted cautiously, as their effectiveness is likely to depend on local implementation factors. Future research should prioritise well-designed trials with standardised intervention frameworks and longer follow-up periods to better understand which components and delivery strategies are most effective in different contexts.

## Supplemental Material

sj-docx-1-smo-10.1177_20503121261444673 – Supplemental material for Multi-pronged biobehavioural intervention strategies for prevention and control of hypertension: A systematic review of education-based community trials Supplemental material, sj-docx-1-smo-10.1177_20503121261444673 for Multi-pronged biobehavioural intervention strategies for prevention and control of hypertension: A systematic review of education-based community trials by Martins Nweke, Nalini Govender, Bernard Appiah and Julian Pillay in SAGE Open Medicine

sj-docx-2-smo-10.1177_20503121261444673 – Supplemental material for Multi-pronged biobehavioural intervention strategies for prevention and control of hypertension: A systematic review of education-based community trials Supplemental material, sj-docx-2-smo-10.1177_20503121261444673 for Multi-pronged biobehavioural intervention strategies for prevention and control of hypertension: A systematic review of education-based community trials by Martins Nweke, Nalini Govender, Bernard Appiah and Julian Pillay in SAGE Open Medicine

sj-docx-3-smo-10.1177_20503121261444673 – Supplemental material for Multi-pronged biobehavioural intervention strategies for prevention and control of hypertension: A systematic review of education-based community trials Supplemental material, sj-docx-3-smo-10.1177_20503121261444673 for Multi-pronged biobehavioural intervention strategies for prevention and control of hypertension: A systematic review of education-based community trials by Martins Nweke, Nalini Govender, Bernard Appiah and Julian Pillay in SAGE Open Medicine

sj-docx-4-smo-10.1177_20503121261444673 – Supplemental material for Multi-pronged biobehavioural intervention strategies for prevention and control of hypertension: A systematic review of education-based community trials Supplemental material, sj-docx-4-smo-10.1177_20503121261444673 for Multi-pronged biobehavioural intervention strategies for prevention and control of hypertension: A systematic review of education-based community trials by Martins Nweke, Nalini Govender, Bernard Appiah and Julian Pillay in SAGE Open Medicine

sj-docx-5-smo-10.1177_20503121261444673 – Supplemental material for Multi-pronged biobehavioural intervention strategies for prevention and control of hypertension: A systematic review of education-based community trials Supplemental material, sj-docx-5-smo-10.1177_20503121261444673 for Multi-pronged biobehavioural intervention strategies for prevention and control of hypertension: A systematic review of education-based community trials by Martins Nweke, Nalini Govender, Bernard Appiah and Julian Pillay in SAGE Open Medicine

sj-docx-6-smo-10.1177_20503121261444673 – Supplemental material for Multi-pronged biobehavioural intervention strategies for prevention and control of hypertension: A systematic review of education-based community trials Supplemental material, sj-docx-6-smo-10.1177_20503121261444673 for Multi-pronged biobehavioural intervention strategies for prevention and control of hypertension: A systematic review of education-based community trials by Martins Nweke, Nalini Govender, Bernard Appiah and Julian Pillay in SAGE Open Medicine
