# Supplementary material for: Multi-pronged biobehavioural intervention strategies for prevention and control of hypertension: A systematic review of education-based community trials
Source: SAGE Open Med. 2026 May 10;14:20503121261444673. doi: 10.1177/20503121261444673 (PMC13168719; doi:10.1177/20503121261444673)
Supplement: sj-docx-2-smo-10.1177_20503121261444673 – Supplemental material for Multi-pronged biobehavioural intervention strategies for prevention and control of hypertension: A systematic review of education-based community trials [file sj-docx-2-smo-10.1177_20503121261444673.docx]

**Supplementary File 2: Study and Demographic Characteristics**

| Author & year | Type of HTN | Diagnostic criteria | Age (mean/median/range) | % Female | Study design | Setting/ethnicity | Prospective or Retrospective | Sampling technique | Sample size | Follow period | Country |
| --- | --- | --- | --- | --- | --- | --- | --- | --- | --- | --- | --- |
| Bunjitpimol & Somrongthong [47] | Hypertension | SBP >140 mmHg and/or DBP >90 mmHg | Mean 52.56  years | 57.4% | RCT | Private hospitals in Bangkok, Thailand; Thai ethnicity | Prospective | Purposive selection | 68  Interv. (34)  Contr. (34) | - | Thailand |
| Cicolini et al. [45] | Hypertension | SBP ≥ 140 or DBP ≥ 90 mmHg or on treatment | Mean 59.0 ± 14.5 years; | 49% | Randomized Controlled Trial | Primary care, Italy | Prospective | Random allocation | 203  Interv. (102)  Contr. (101) | 6 months | Italy |
| Elgendy et al. [48] | Not specifically classified | Not specified | Mean 58.80 ± 10.04 years | 72% | Single group pre-post | Two  hospitals in the governorates of Alexandria and Kafr El  Shiekh | Prospective | Random sampling | 300*  Post- (300)  Pre- (300) | 6 months | Egypt |
| Ferrara et al. [46] | Arterial hypertension (not further subclassified) | Stable BP with drug therapy in prior 6 months; baseline SBP ≥132 mm Hg, DBP ≥83 mm Hg | Mean: 56.4 ± 9.5 | 47.9% | RCT | Outpatient clinic, Italy | Prospective | Random sampling | 188  Interv. (94)  Contr. (94) | 2 years | Italy |
| Gabiola et al. [49] | Not specifically classified | Range of 130–159/90–99 mmHg,  based on the Joint National Committee on Prevention,  Detection, Evaluation, and Treatment of High Blood pres  sure (JNC7) criteria for stage 1 hypertension and pre-hyper  tension. | Median: 49.5 (40.75, 56.38) | 75.4% | Community trial | Two main communities (barangays) in  District V of Manila, Philippines | Prospective | Convenience sampling | 159  Interv. (82)  Contr. (77) | 6 months | Philippines |
| Hazratigonbad et al. [41] | Primary hypertension | Not specified | Mean age: 49.46 (7.09) | 71.8% | RCT | Public health care centers in Tehran, Iran | Prospective | Convenience sampling | 94  Interv. (45)  Contr. (49) | 2 months | Iran |
| Hunt et al. [37] | Mild Hypertension | Last BP 140–159/90–99 mmHg from EMR | Mean age: 69.2 years (SD 12.4) | 57% | Prospective, Randomized, Controlled, Single-Blind Trial | Primary care clinics in Portland, Oregon; mostly white ethnicity | Prospective | Random selection | 312  Interv. (162)  Contr. (150) | 1 year (±3 months) | USA |
| Jafari et al. [42] | Hypertension (not further specified) | Not specified | Mean age: 57 ± 6.5 years | Not stated | RCT | An educational hospital in Isfahan, Iran, | Prospective | Convenience sampling | 60  Interv. (30)  Contr. (30) | 3 weeks | Iran |
| James et al. [50] | Not specifically classified; general hypertension cases | Clinically diagnosed hypertension receiving treatment for ≥2 months; SBP ≥140 or DBP ≥90 mmHg (or ≥150/90 mmHg for patients ≥80 years) | Mean: 67.36±12.97 (intervention), 64.76±13.18 (control) | 57.6% | Non-randomised controlled trial | Kamjong block, Kamjong district, Manipur, India | Prospective | Convenience sampling with lottery method for group allocation | 66  Interv. (33)  Contr. (33) | 3 months | India |
| Johnson et al. [38] | Uncontrolled hypertension | SBP ≥140 mmHg or DBP ≥90 mmHg; for diabetics, SBP ≥130 mmHg or DBP ≥80 mmHg | Mean age: 56.75 (14.1) | 66.05% | Randomized controlled trial (4-arm) | USA; predominantly African American population | Prospective | Convenience sampling within clinical practices | 552  Interv. (203)  Contr. (249) | 6 months | USA |
| Khani Jeihooni et al [43] | Hypertension (not further subtyped) | Use of antihypertensives | Mean age: 53 (14) | 55% | Randomized controlled clinical trial | Fasa Diabetes Center, Iran | Prospective | Simple random sampling | 300  Interv. (150)  Contr. (150) | 3 months | Iran |
| Kim et al., [39] | Not specified | SBP ≥140 mmHg and/or DBP ≥90 mmHg or on antihypertensive medication | Mean age = 70.9±5.3 years | 69.9% | Clinical controlled trial | Community-based; Korean Americans | Prospective | Random sampling | 369  Interv. (184)  Contr. (185) | 18 months | USA |
| Kordvarkane et al. [44] | Hypertension (not subtyped) | SBP ≥140 mmHg or DBP ≥90 mmHg or use of antihypertensive medications | Mean age: 54.53 (8.19) | 60.83% | RCT | Farshchian Hospital clinic in Hamadan | Prospective | Random sampling | 68  Interv. (33)  Contr. (35) | 3 months | Iran |
| Kwiringira et al. [51] | Hypertension (not subtyped) | Systolic BP (SBP)≥140 mm Hg and/or diastolic BP (DBP)≥90 mmHg | 18 >50 | 59.6% | Quasi-experimental study | Kalangala and Buvuma Island  Districts of Lake Victoria, Uganda | Prospective | Random sampling | 2016*  Post- (2016)  Pre- (2016) | 3 months | Uganda |
| Ma et al. [40] | Hypertension (not subtyped) | Systolic (S)BP≥140 mmHg  or diastolic (D)BP≥90 mmHg, or current use of antihypertensive  medication | - | 70.5% | RCT | Filipino Americans | Prospective | Random sampling | 71  Interv. (47)  Contr. (24) | 3 months | USA |
| Thapa et al. [53] | Not specified | Mean SBP of 140 mm Hg or higher, or mean DBP of 90 mm Hg or higher, or being on regular antihypertensive therapy | 50.05 (8.45) | 59.1% | Cluster Randomised Trial | Lekhnath Municipality, Kaski, Gandaki  Province, Nepal | Prospective | Cluster random sampling | 395  Interv. (168)  Contr. (227) | 60 months | Nepal |
| Lai et al. [52] | Not specified | SBP ≥140 mmHg and/or DBP ≥90 mmHg or antihypertensive use | Mean ≈ 55 years | Not reported | Randomized controlled trial | Community health centers | Prospective | Random allocation | 300 (Interv. 150; Contr. 150) | 6 months | China |

Pre: pre-intervention; post: post-intervention. Note: throughout the analysis, for the single group design, post-intervention scores were treated as intervention arm while the pre-was treated as the control arm.
