# Supplementary material for: Multi-pronged biobehavioural intervention strategies for prevention and control of hypertension: A systematic review of education-based community trials
Source: SAGE Open Med. 2026 May 10;14:20503121261444673. doi: 10.1177/20503121261444673 (PMC13168719; doi:10.1177/20503121261444673)
Supplement: sj-docx-3-smo-10.1177_20503121261444673 – Supplemental material for Multi-pronged biobehavioural intervention strategies for prevention and control of hypertension: A systematic review of education-based community trials [file sj-docx-3-smo-10.1177_20503121261444673.docx]

# Supplementary File 3: Risk of bias for NRCT using the ROBINS-I Risk-of-Bias Assessment Tool

| Study | Confounding | Selection of participants | Classification of interventions | Deviations from intended interventions | Missing data | Measurement of outcomes | Selection of reported results | Overall ROBINS-I judgment | Overall risk of bias rating |
| --- | --- | --- | --- | --- | --- | --- | --- | --- | --- |
| Kwiringira et al. [51] | Moderate | Low | Low | Low | Low | Moderate | Low | Moderate | Moderate |
| Elgendy et al. [48] | Serious | Moderate | Low | Serious | Moderate | Moderate | Moderate | Serious | Serious |
| James et al. [50] | Moderate | Low | Low | Moderate | Low | Moderate | Low | Moderate | Moderate |
| Gabiola et al. [49] | Serious | Low | Low | Moderate | Low | Moderate | Low–Moderate | Serious | Serious |
