# Supplementary material for: Multi-pronged biobehavioural intervention strategies for prevention and control of hypertension: A systematic review of education-based community trials
Source: SAGE Open Med. 2026 May 10;14:20503121261444673. doi: 10.1177/20503121261444673 (PMC13168719; doi:10.1177/20503121261444673)
Supplement: sj-docx-4-smo-10.1177_20503121261444673 – Supplemental material for Multi-pronged biobehavioural intervention strategies for prevention and control of hypertension: A systematic review of education-based community trials [file sj-docx-4-smo-10.1177_20503121261444673.docx]

Supplementary File 4


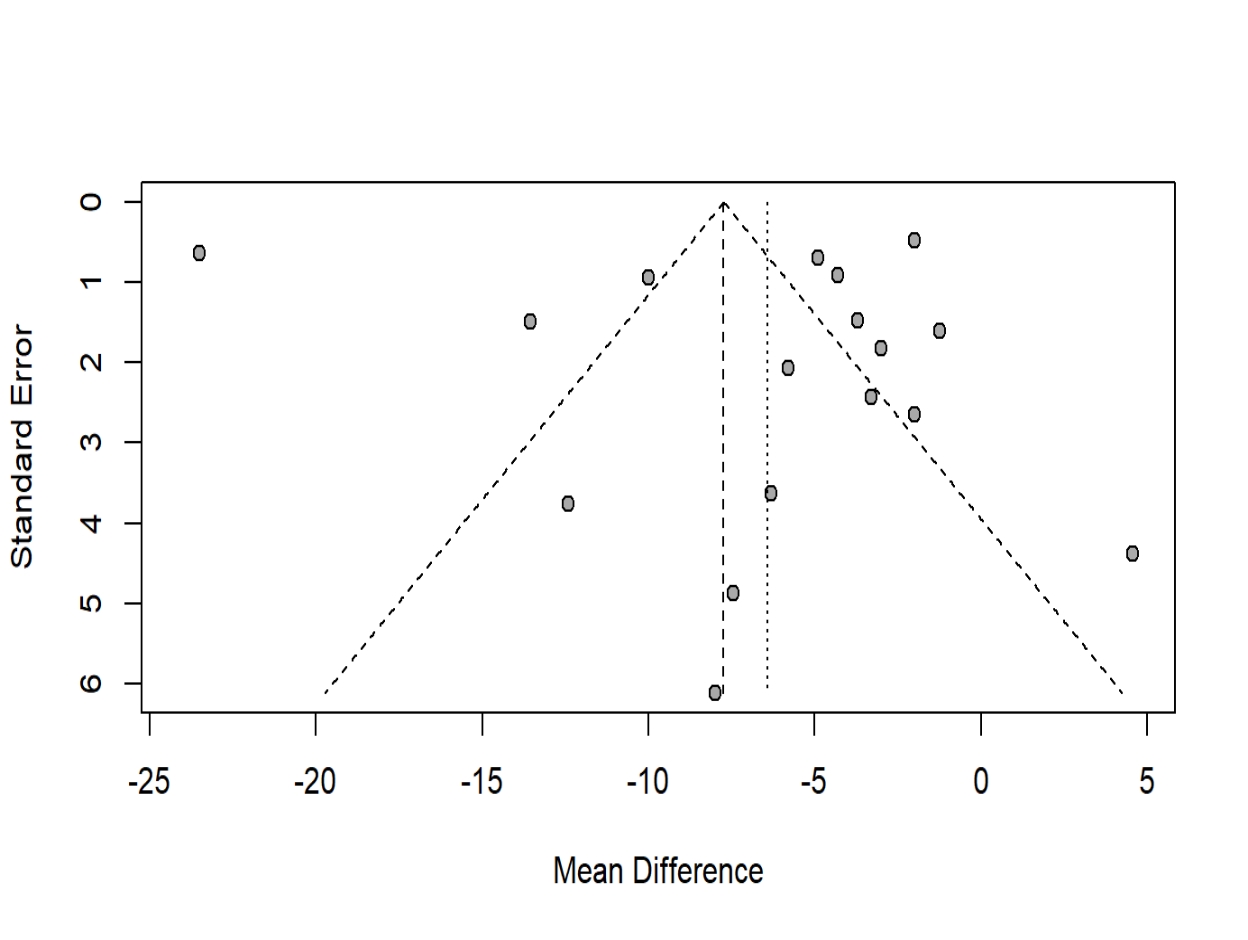


Fig. 8 Funnel plot displaying likelihood for publication bias in studies included in the analysis of the effect of education-based bio-behavioural interventions for blood pressure control (SBP)


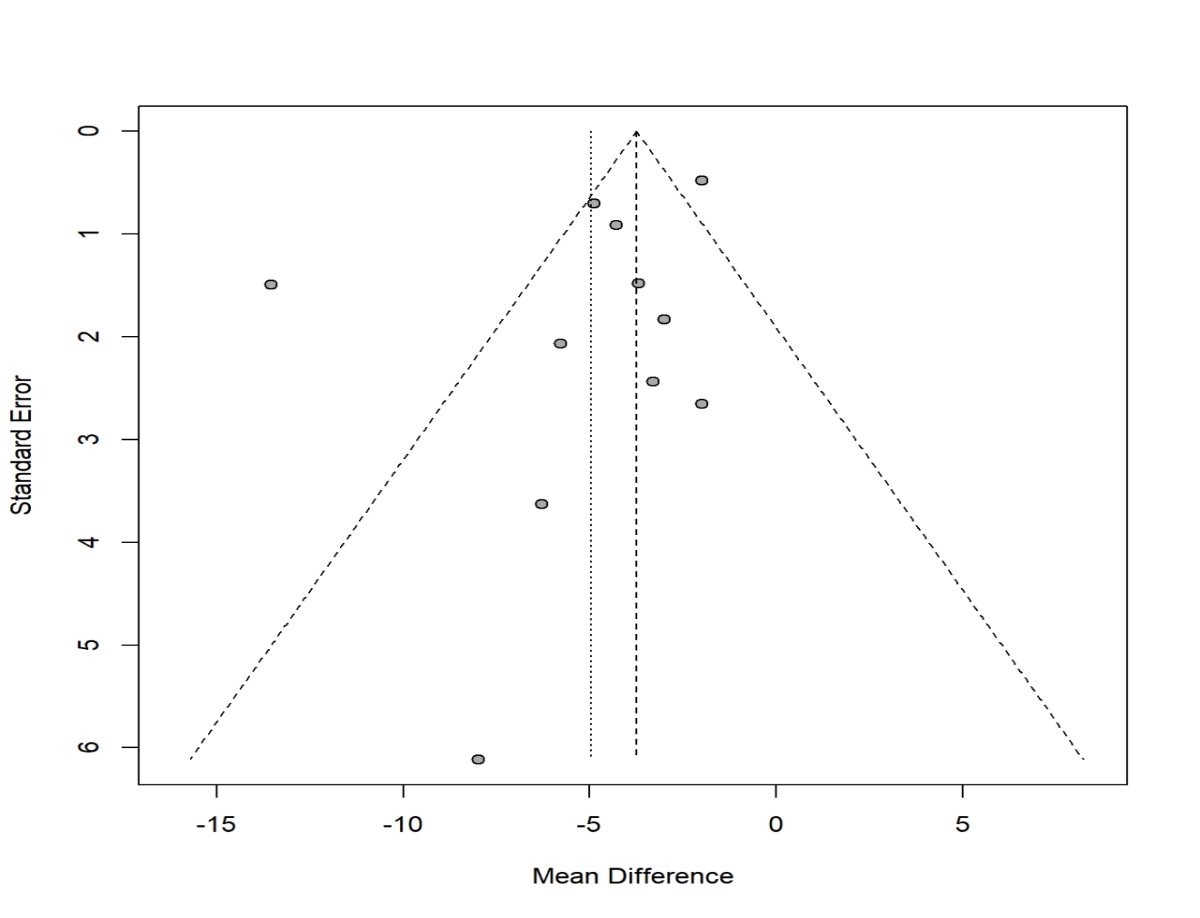


Fig. 9 Funnel plot showing the likelihood of publication bias in the pooled estimate of MBBI for systolic BP when limited to intervention category 1 (≥ four components)


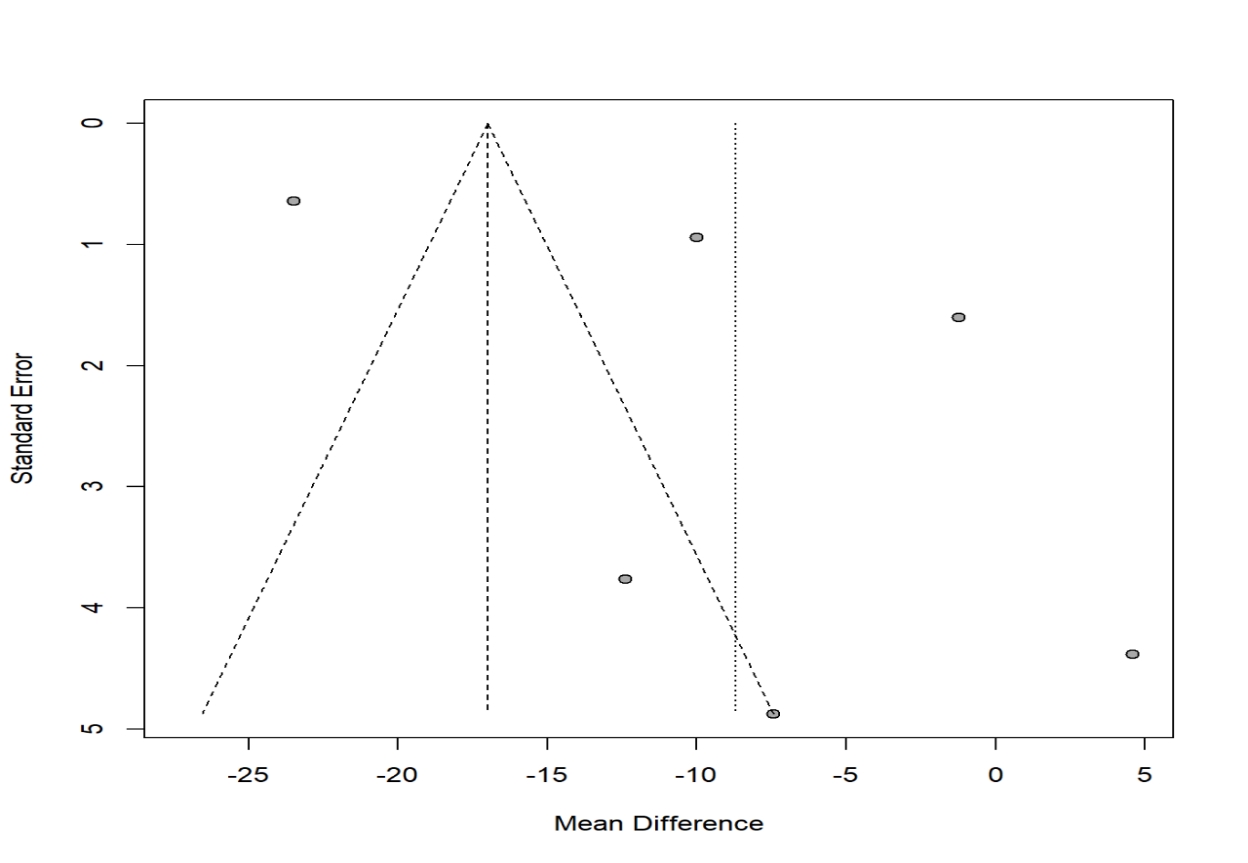


Fig. 10 Funnel plot showing the likelihood of publication bias in the pooled estimate of MBBI for systolic BP when limited to intervention category 1 (two-three components)


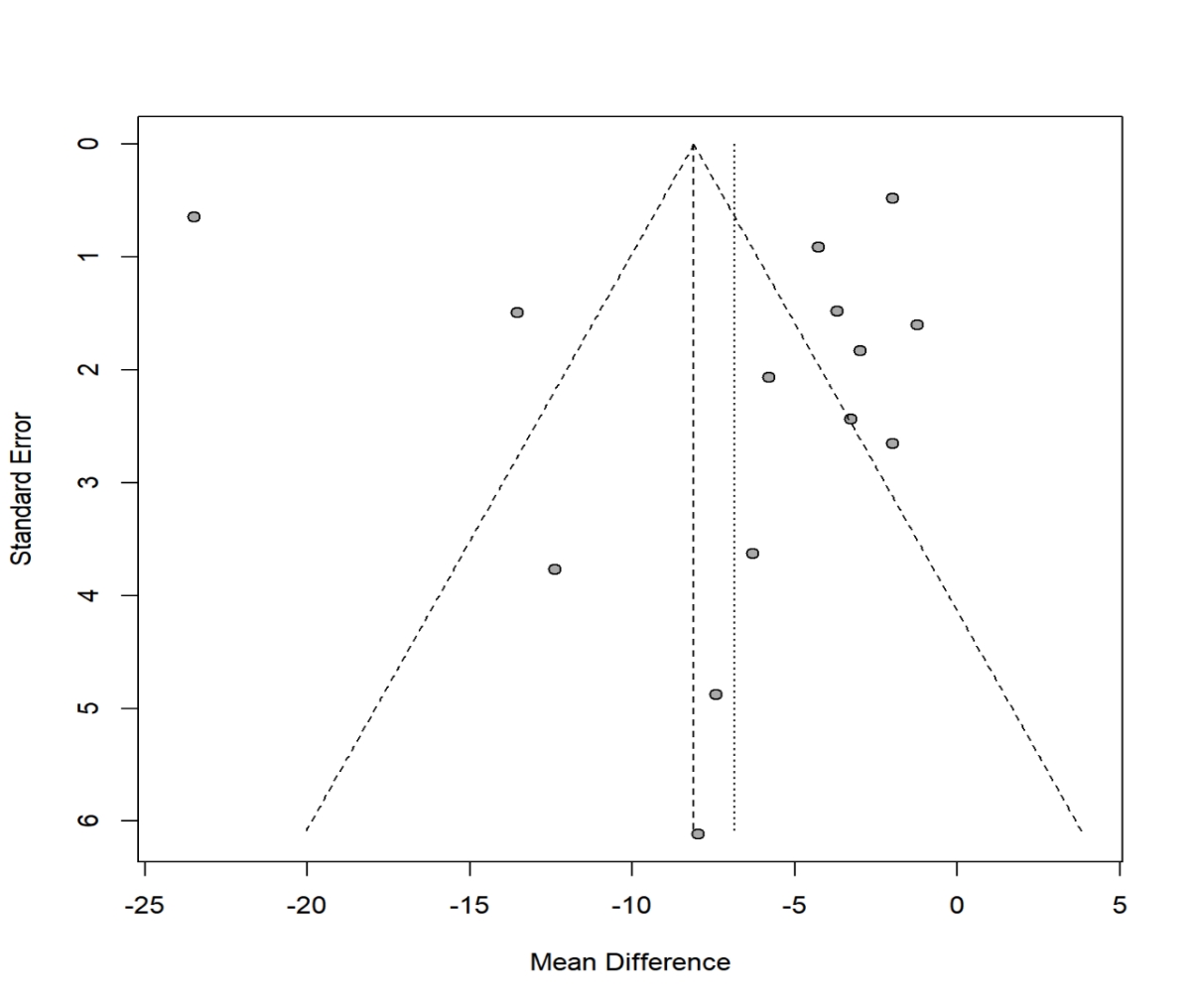


Fig.11 Funnel plot showing the likelihood of publication bias in the pooled estimate of MBBI for systolic BP when limited to study design (RCT)


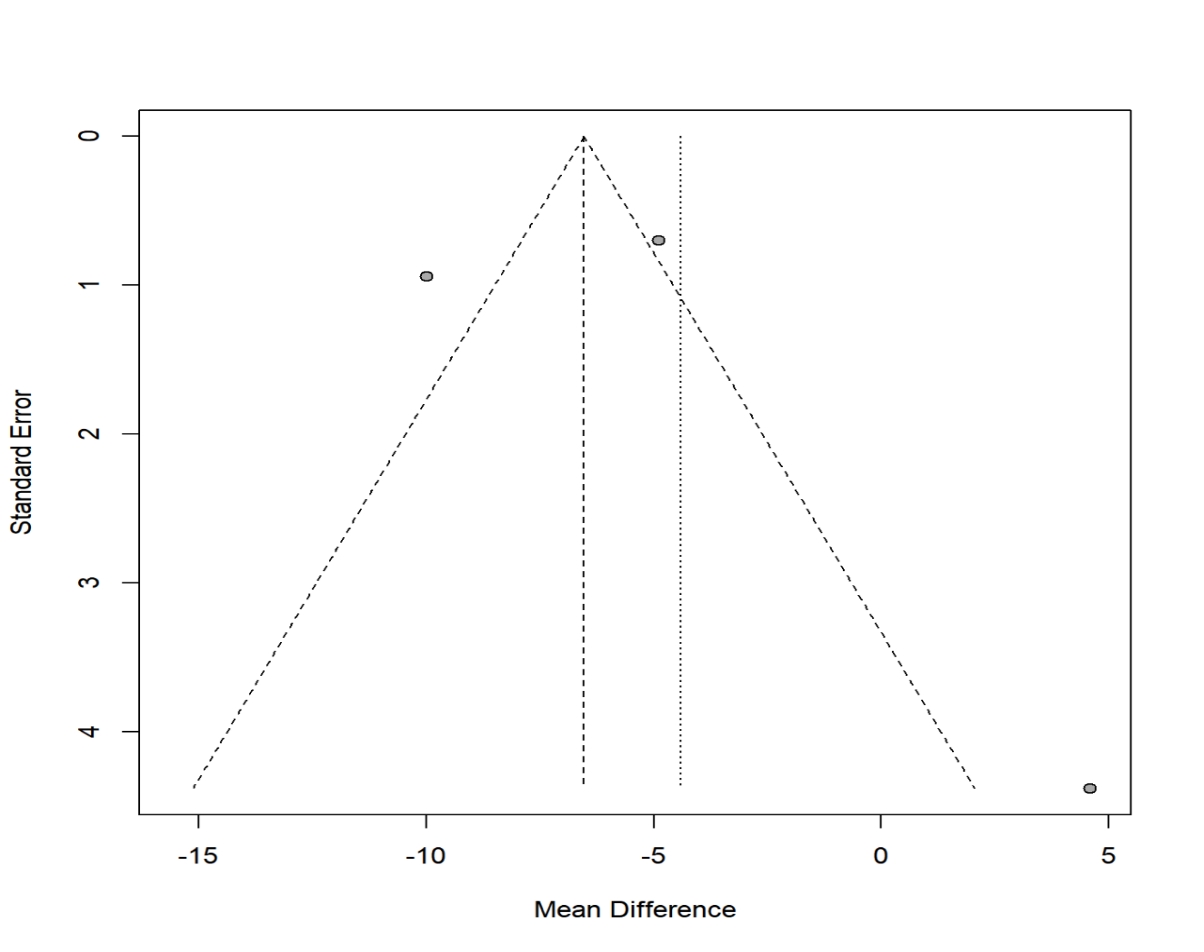


Fig. 12 Funnel plot showing the likelihood of publication bias in the pooled estimate of MBBI for systolic BP when limited to the study design (NRCT).


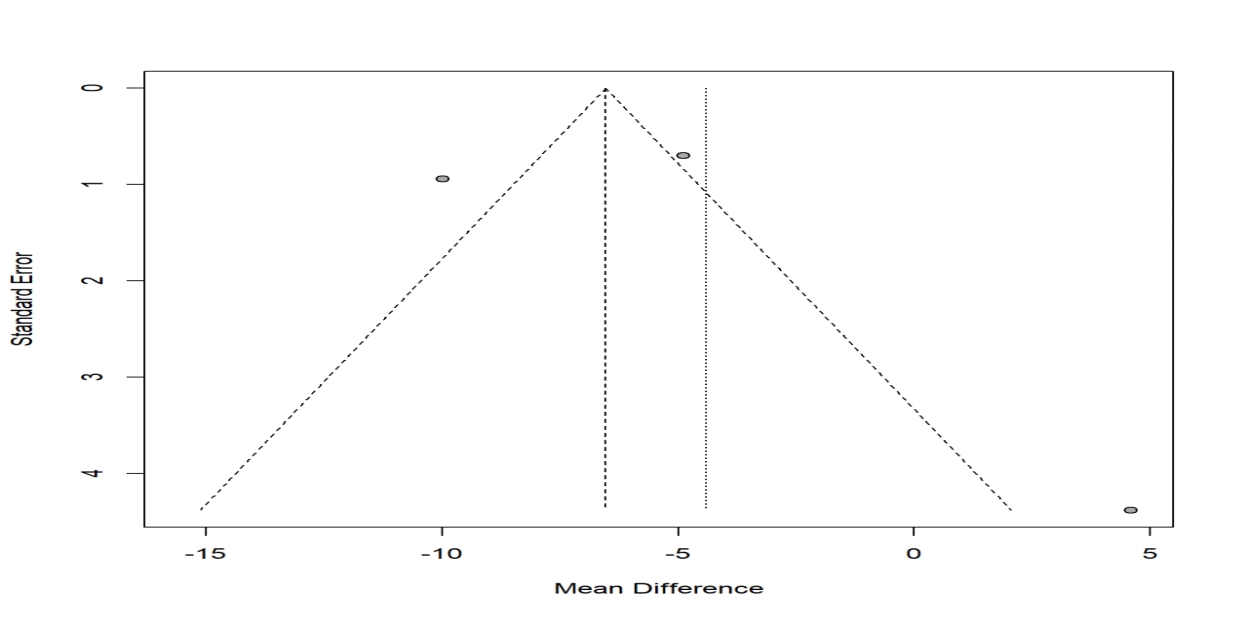


Fig. 13 Funnel plot showing the likelihood of publication bias in the pooled estimate of MBBI for systolic BP when limited to intervention duration (≤ three months)


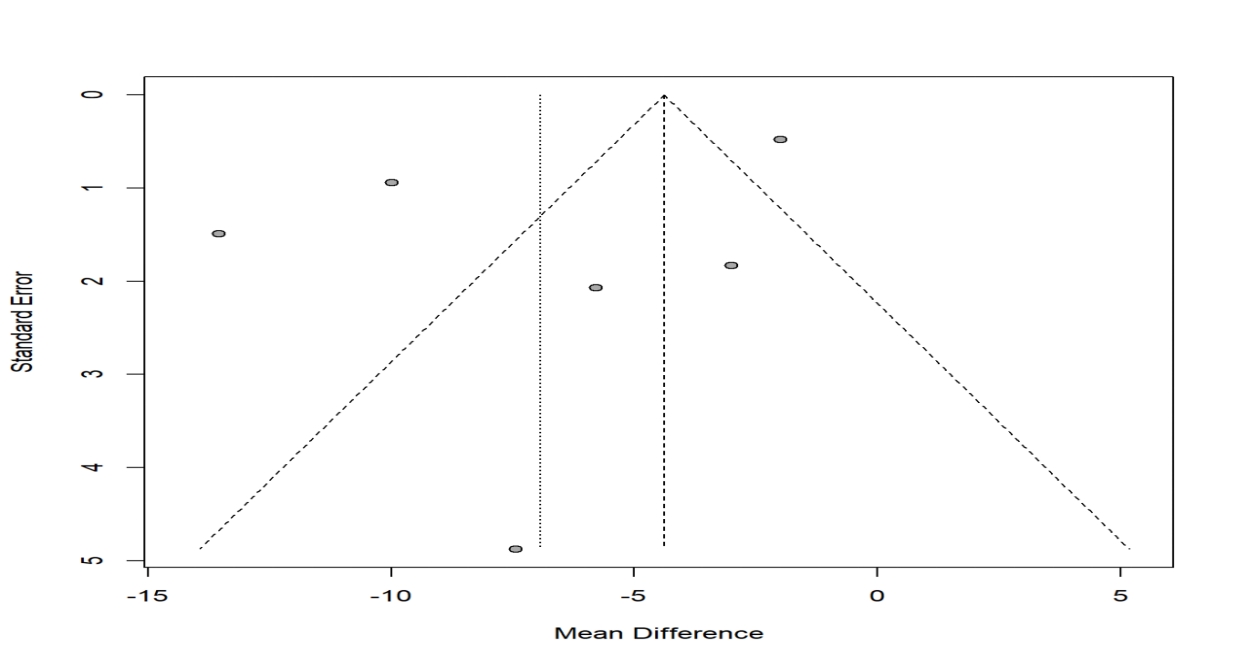


Fig. 14 Funnel plot showing the likelihood of publication bias in the pooled estimate of MBBI for systolic BP when limited to intervention duration (≥ six months)


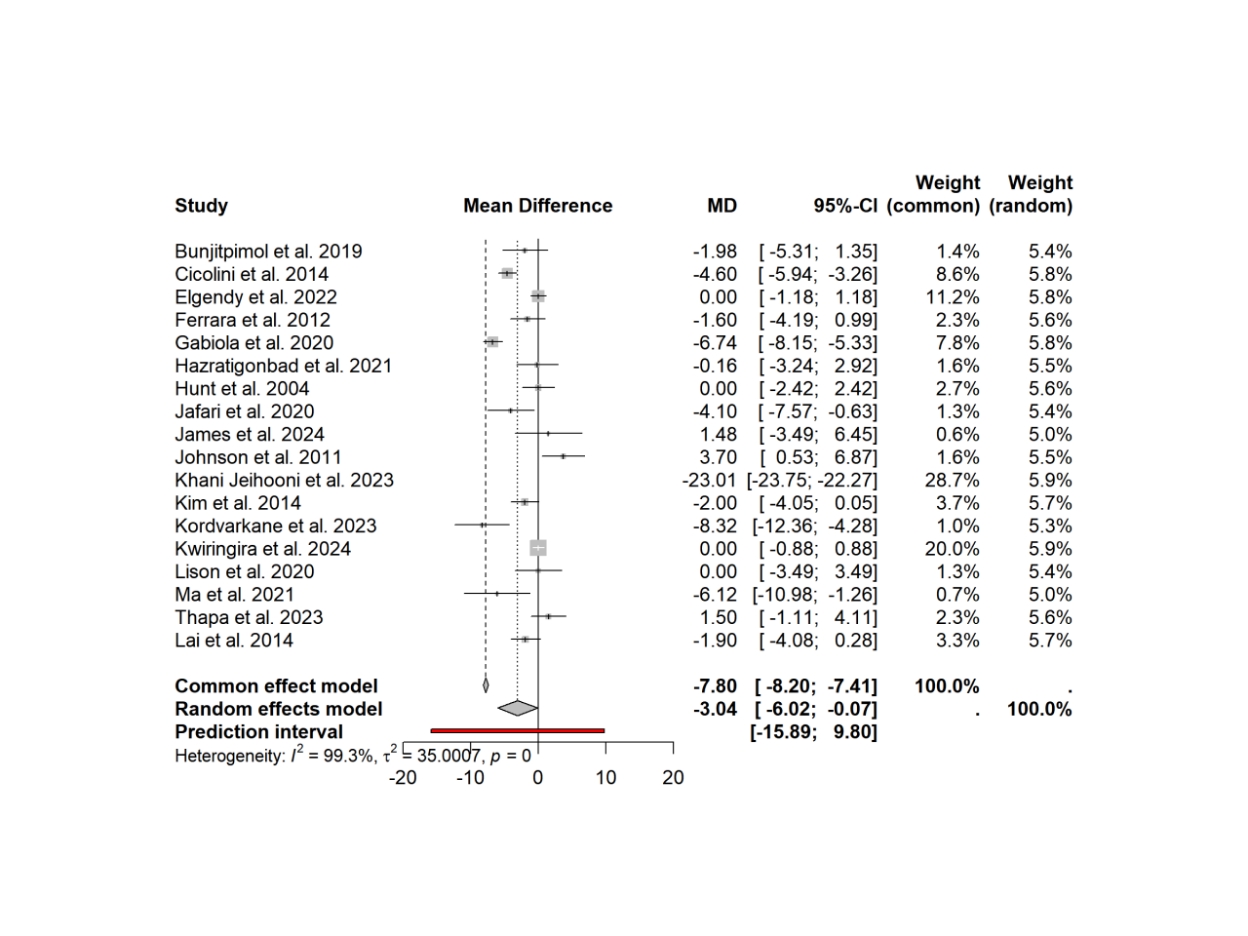


Fig. 15 Forest plot displaying the efficacy of education-based BBI for diastolic blood pressure


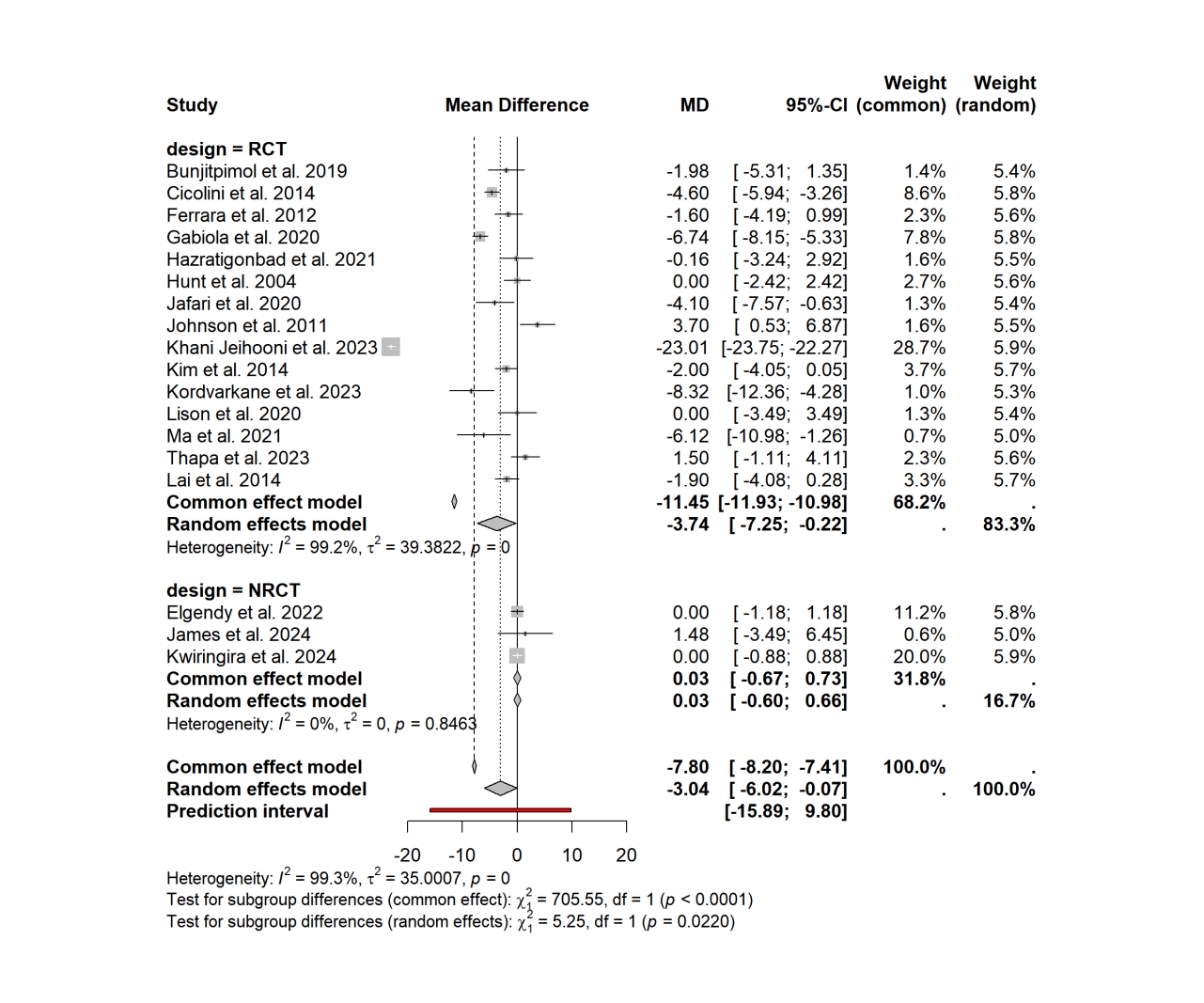


Fig.16 Forest plot sub-group analysis displaying the impact of variation in study design on the pooled estimate


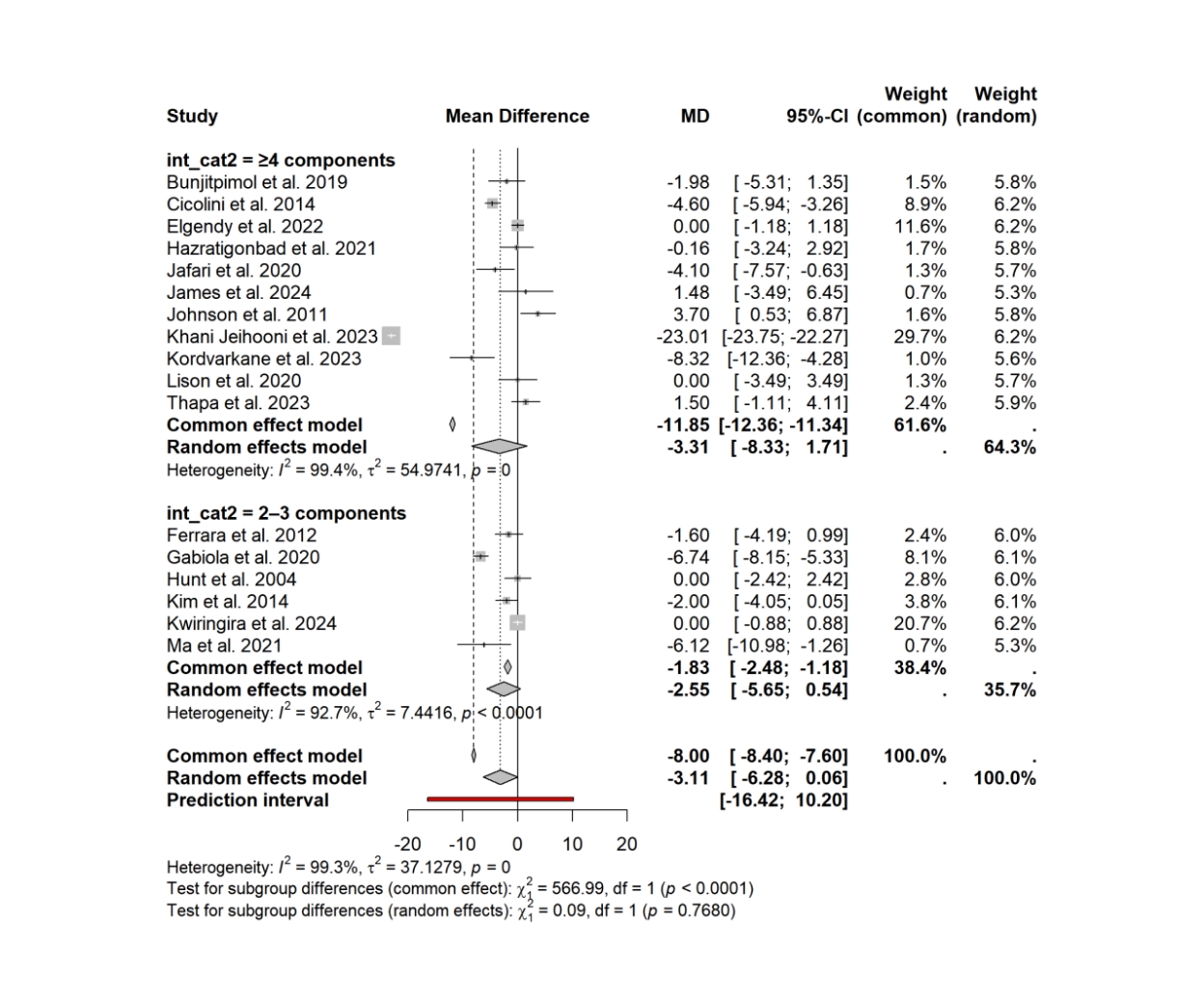


Fig. 17 Forest plot sub-group analysis displaying the impact of variation in intervention category on the pooled estimate


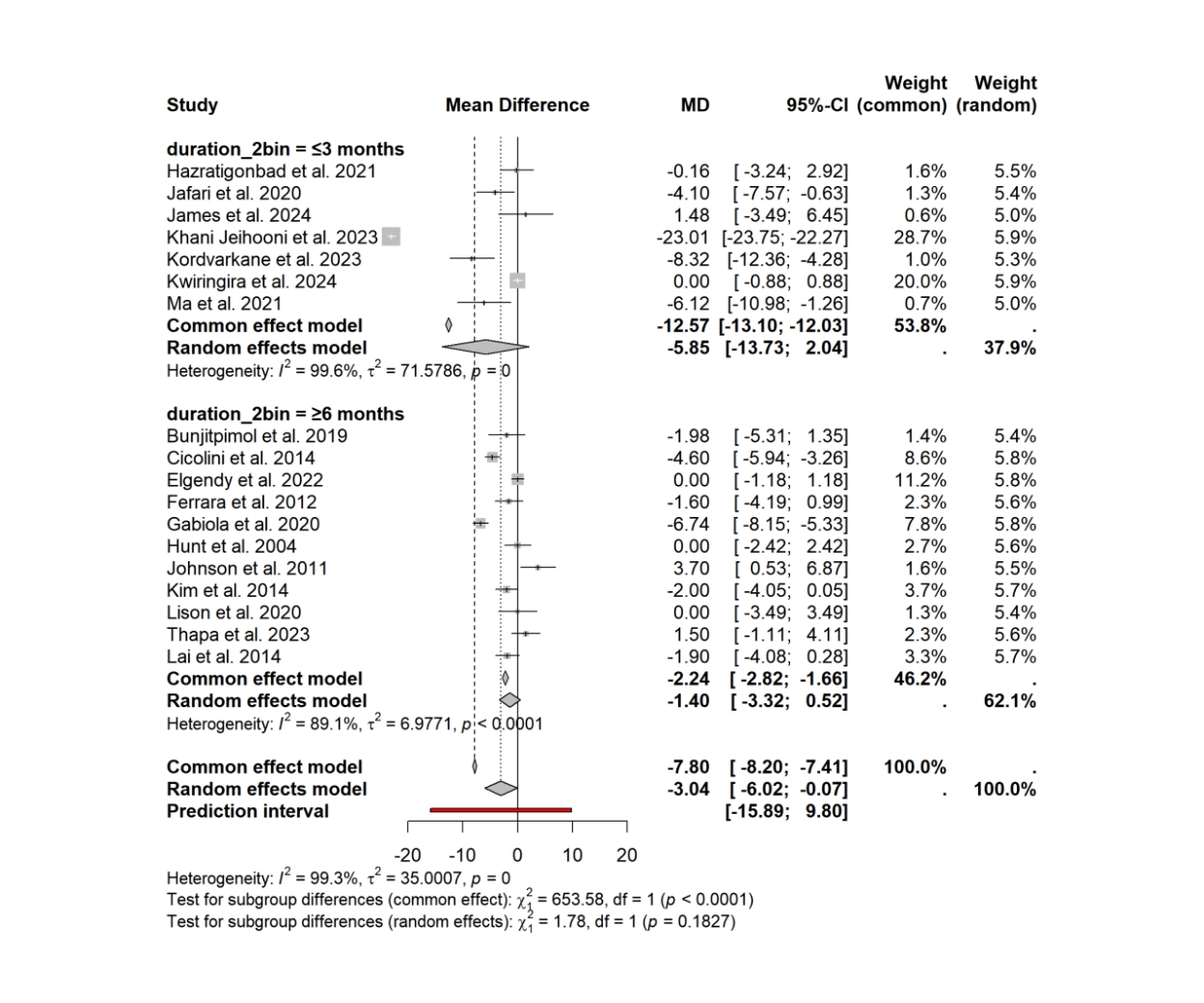


Fig. 18 Forest plot sub-group analysis displaying the impact of variation in intervention duration on the pooled estimate


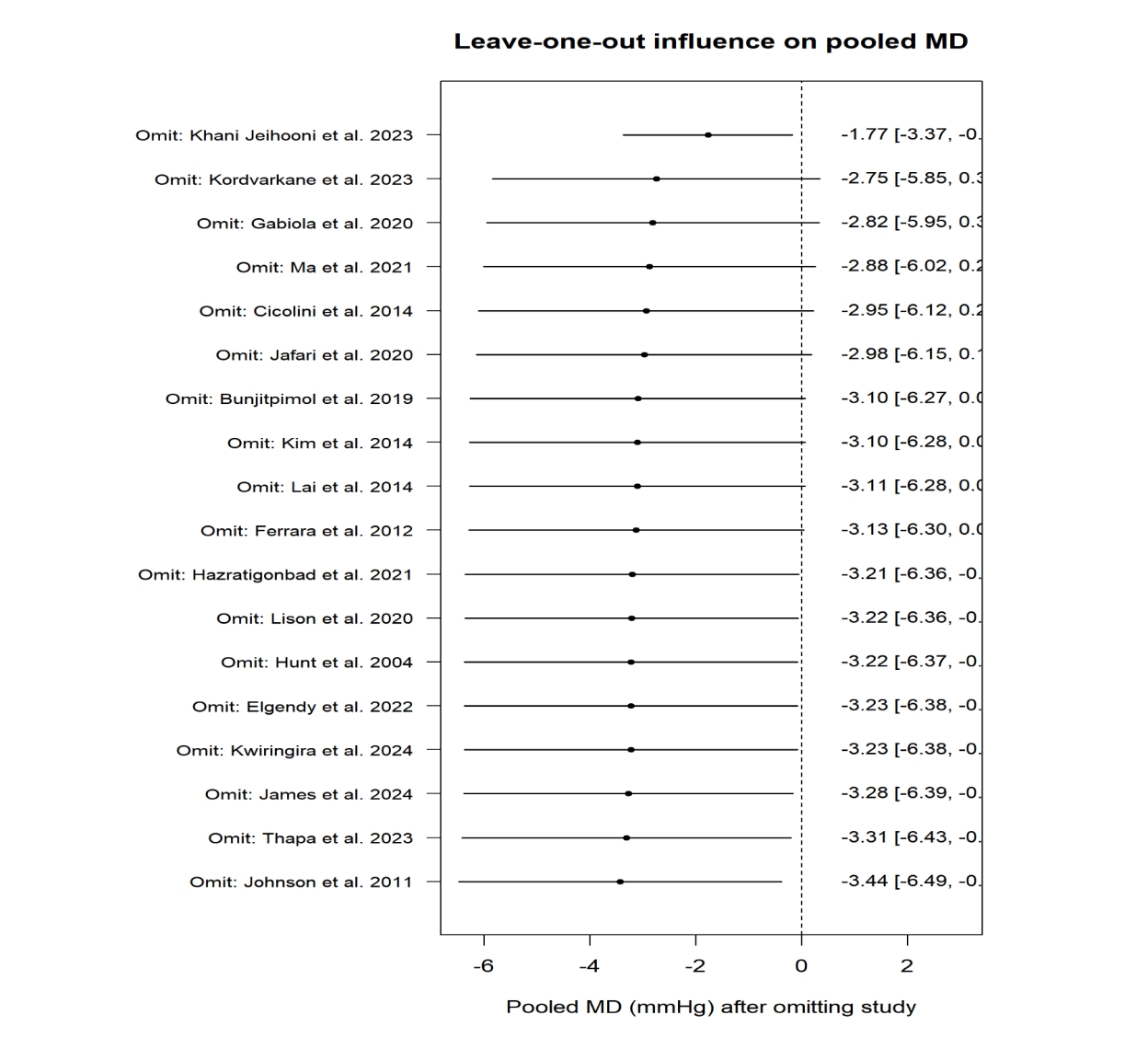


Fig. 19 Forest plot showing the influence of the leave-one-out analyses on pooled MD


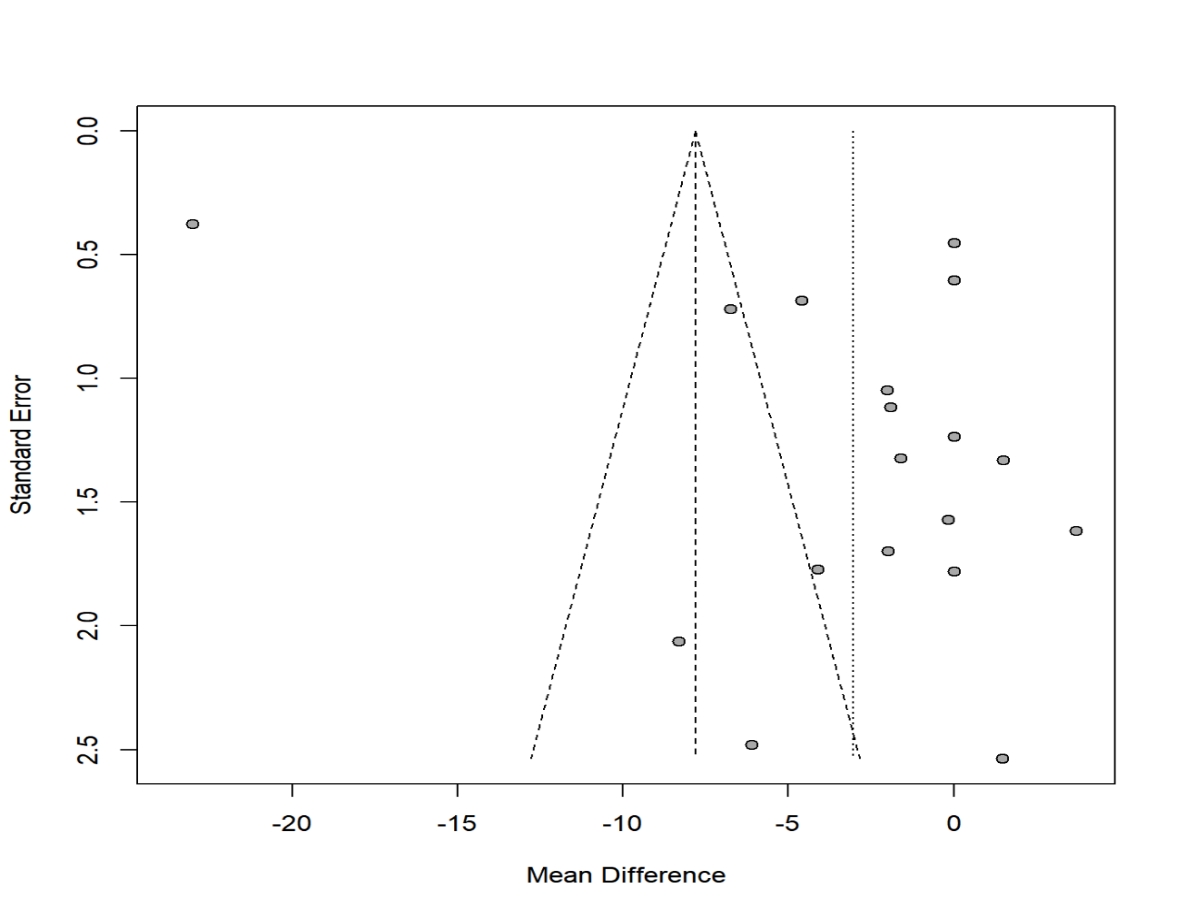


Figure 20 Funnel plot displaying likelihood for publication bias in studies included in the analysis of the effect of education-based bio-behavioural interventions for blood pressure control (DBP).


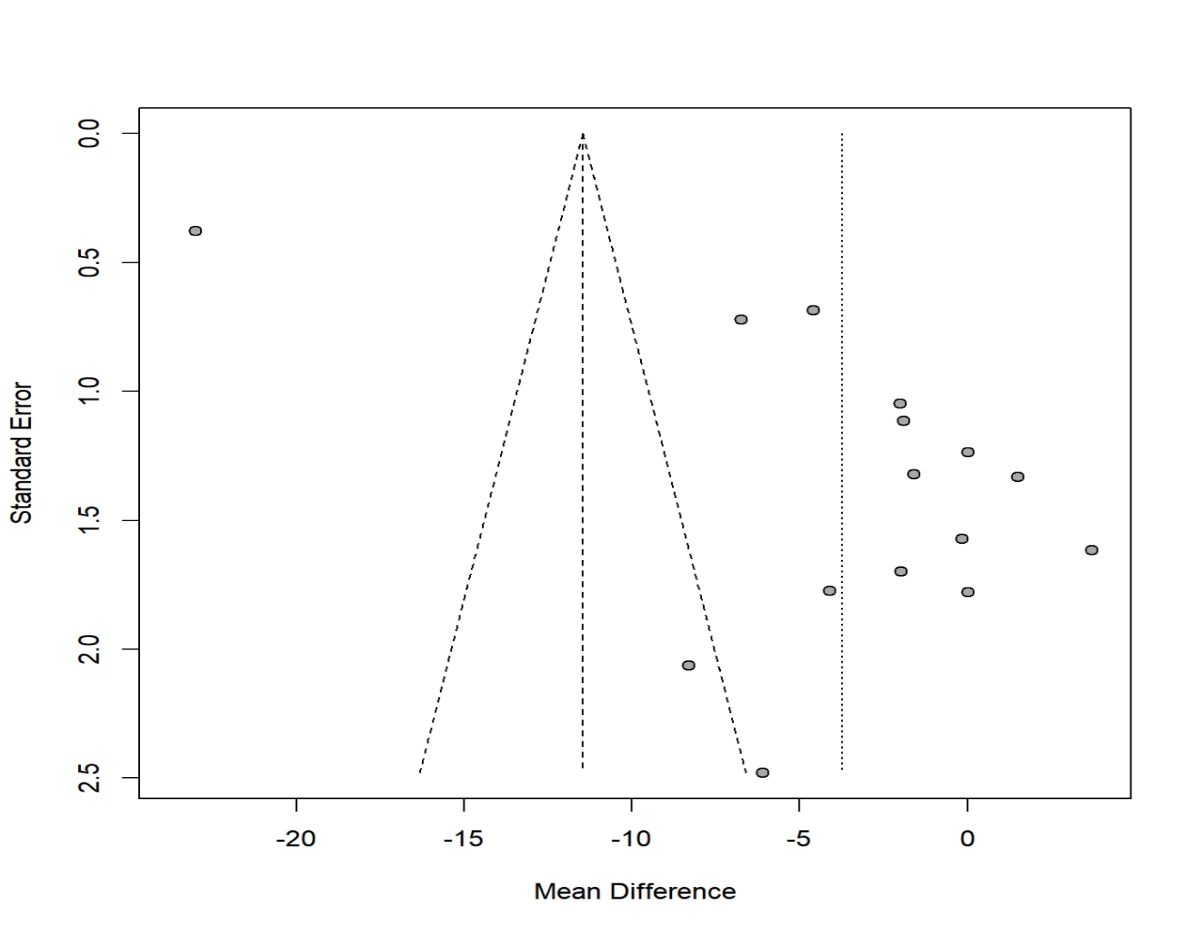
 Fig. 21 Funnel plot showing the likelihood of publication bias in the pooled estimate of MBBI for diastolic BP when limited to study design (RCT).


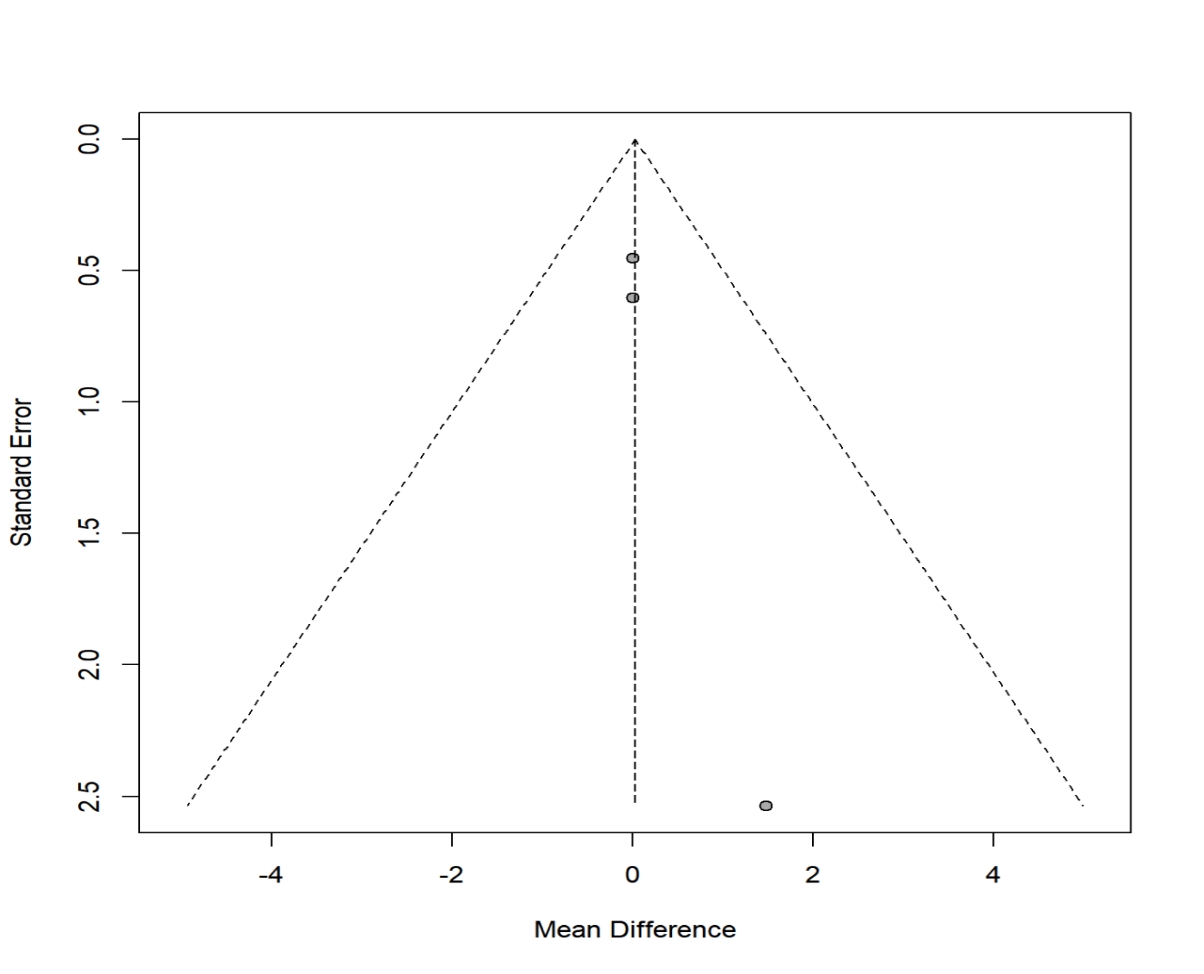
 Fig. 22 Funnel plot showing the likelihood of publication bias in the pooled estimate of MBBI for diastolic BP when limited to study design (NRCT).


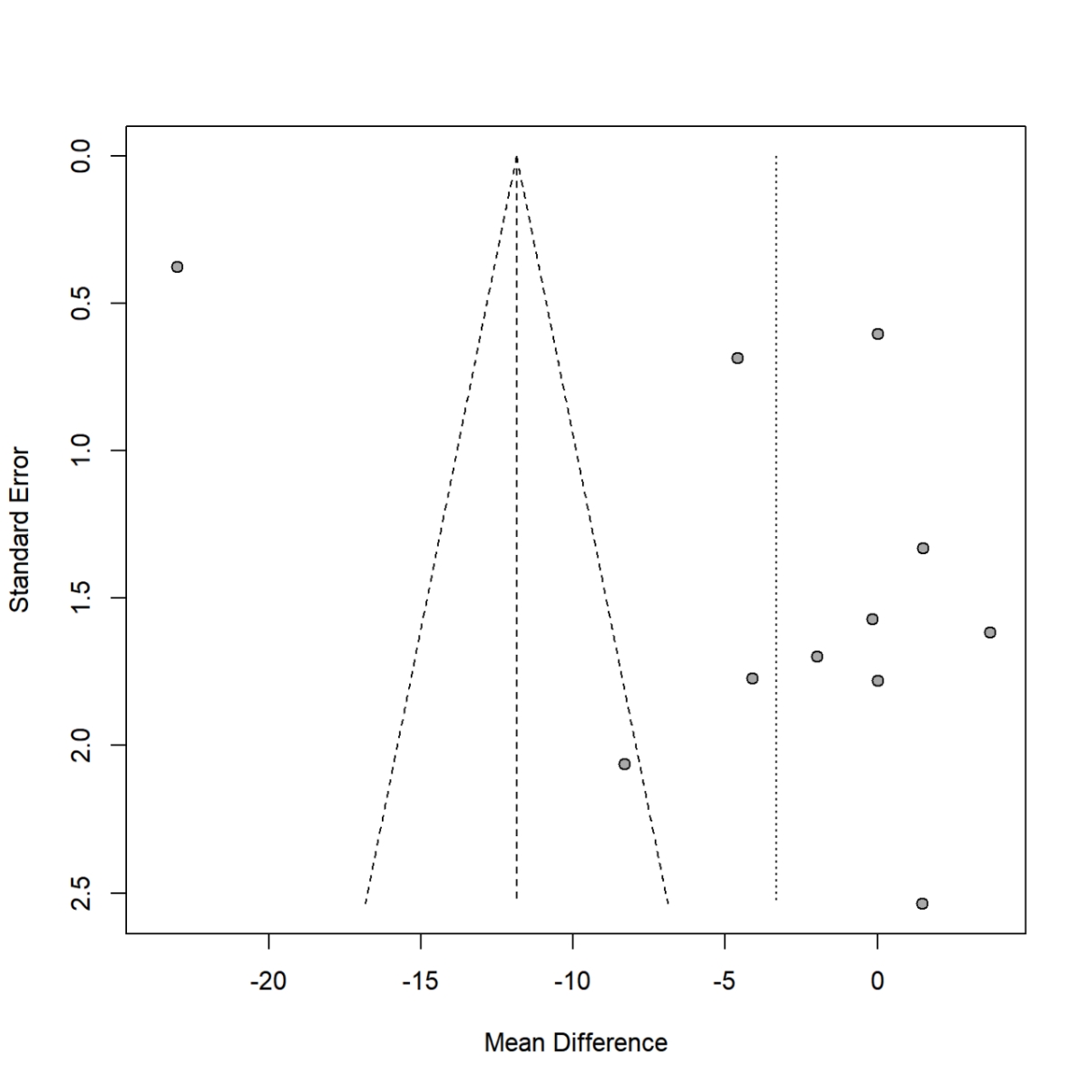
 Fig. 23 Funnel plot showing the likelihood of publication bias in the pooled estimate of MBBI for diastolic BP when limited to intervention category 1.


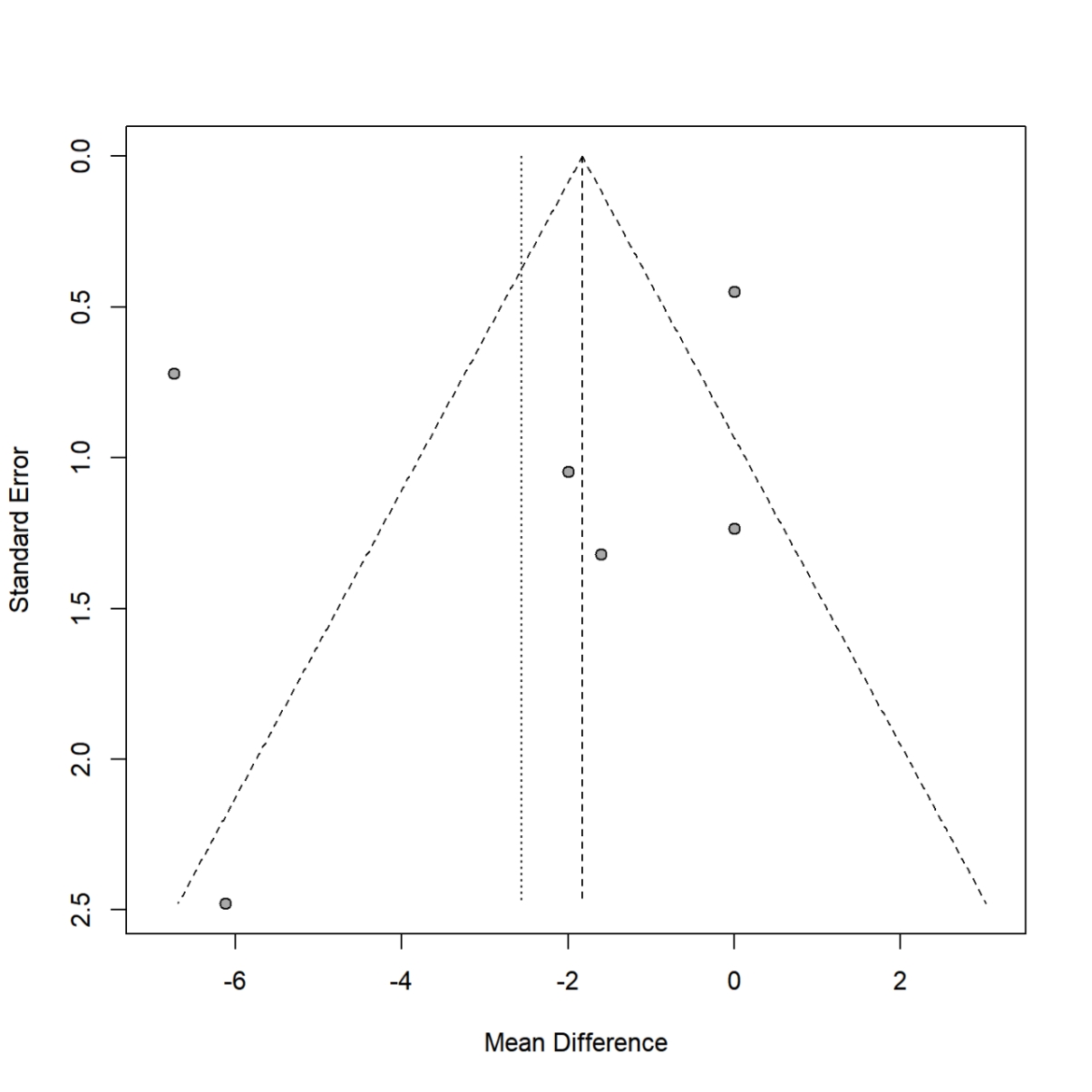


Fig. 24 Funnel plot showing the likelihood of publication bias in the pooled estimate of MBBI for diastolic BP when limited to intervention category 2.


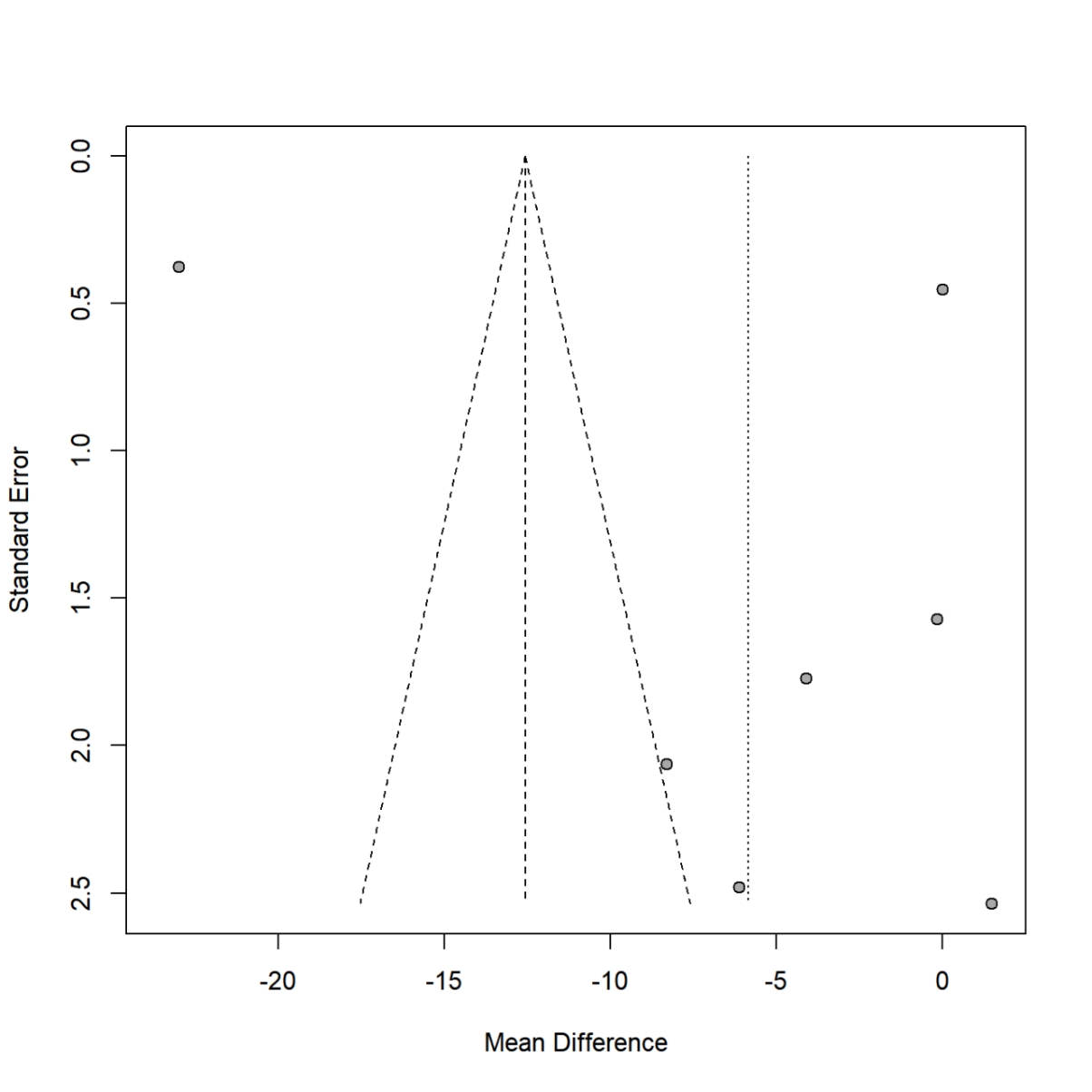


Fig. 25 Funnel plot showing the likelihood of publication bias in the pooled estimate of MBBI for diastolic BP when limited to intervention duration (≤ 3months).


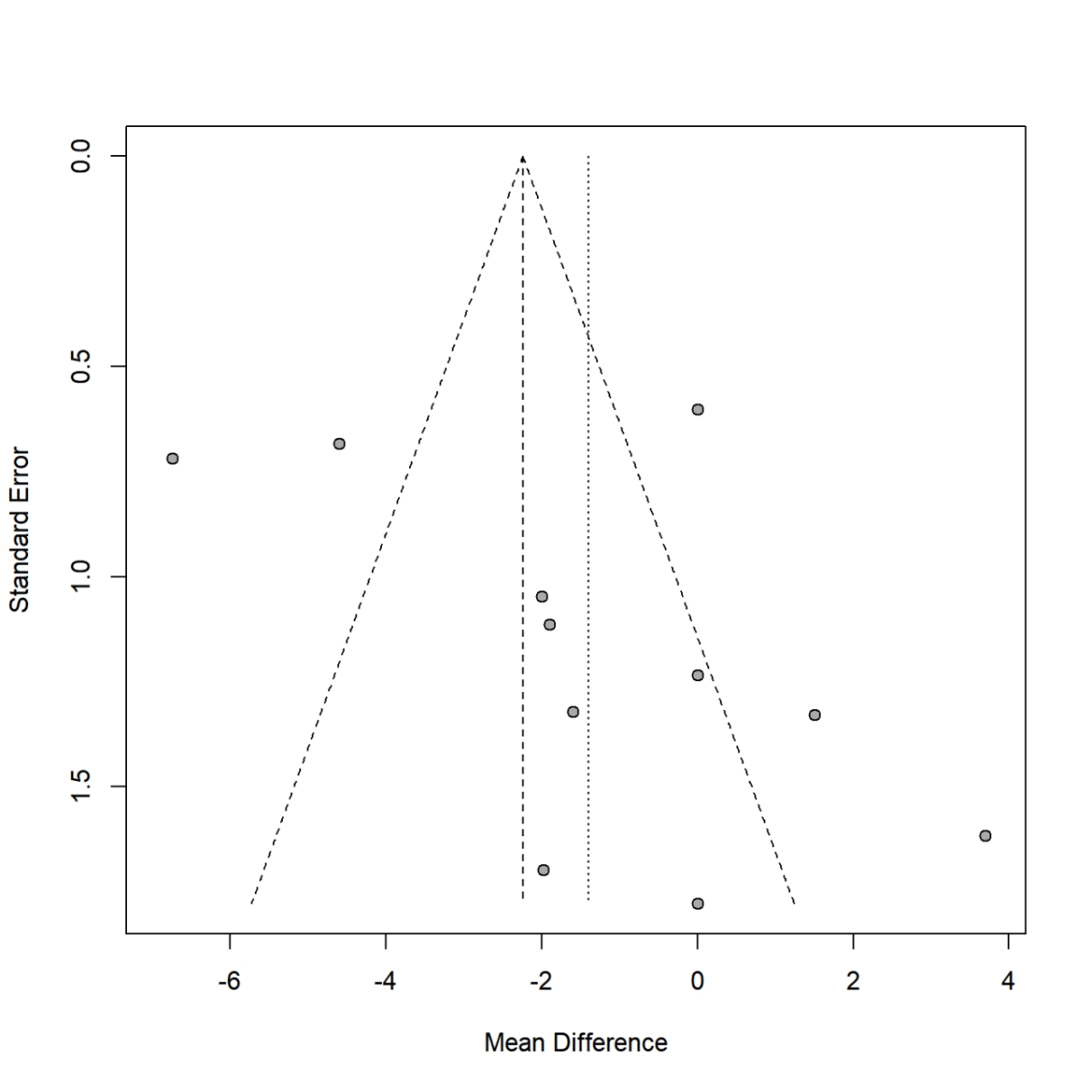


Fig. 26 Funnel plot showing the likelihood of publication bias in the pooled estimate of MBBI for diastolic BP when limited to intervention duration (≥ 6 months).
