# Supplementary material for: Multi-pronged biobehavioural intervention strategies for prevention and control of hypertension: A systematic review of education-based community trials
Source: SAGE Open Med. 2026 May 10;14:20503121261444673. doi: 10.1177/20503121261444673 (PMC13168719; doi:10.1177/20503121261444673)
Supplement: sj-docx-5-smo-10.1177_20503121261444673 – Supplemental material for Multi-pronged biobehavioural intervention strategies for prevention and control of hypertension: A systematic review of education-based community trials [file sj-docx-5-smo-10.1177_20503121261444673.docx]

**Supplementary File 5: Narrative synthesis of the outcome of the multimodal educational interventions**

|  | Intervention description/Mode of delivery | Health professionals involved | Study design & Population | Duration & follow-up period | Sample size & sampling technique | Outcome assessment measure | Age & sex distribution | Effect size & mode of analysis | Setting/  Country |
| --- | --- | --- | --- | --- | --- | --- | --- | --- | --- |
| BP/BP control | | | | | | | | |  |
| Bunjitpimol et al. [47]  [BP: M(SD)] | Exercise + stress management + diet control + alcohol + smoking  MOD: face to face (group sessions) and digital | NS | RCT; Adults aged between 35 to 60 years | 6 months | 68 (Control=34; Intervention=34); Purposive sampling | ns | Mean 52.56 (7.03) years; 57.4% female | SBP: Cohen’s d = -0.33 (-0.81, 0.15)  (Control = 134.17 ± 9.75; Intervention = 130.87 ± 10.32)  DBP: Cohen’s d = -0.28 (-0.76, 0.20)  (Control = 80.65 ± 6.81; Intervention = 78.67 ± 7.19) | Thailand |
| Cicolini et al. [45]  [BP: M(SD)] | Diet + physical activity + smoking + alcohol + obesity + psychological stress. Once weekly for 6 months.  MOD: face to face (group session), emails & phone calls | Nurses | RCT; Hypertensives on active treatment for HTN or SBP  _x0005_≥ 140 mmHg; or  DBP ≥ 90 mmHg | 6 months | 198 (Control=98; Intervention=100); Random sampling | ns | Mean 59.05 (14.45) years; 49% female | SBP: Cohen’s d = 0.49 (0.21, 0.77)  (Control = 143 ± 6; Intervention = 135 ± 8)  DBP: Cohen’s d = -0.95 (-1.24, -0.66)  (Control = 81.0 ± 3.6; Intervention = 76.4 ± 5.8) | Italy |
| Elgendy et al. [48]  [BP: M(SD)] | Weight loss + reduced sodium intake + increased physical activity + cessation of tobacco consumption + lifestyle modifications  MOD: face to face | NS | Quasi - experiment; Grade I hypertensive patients | 6 months | 300; Random sampling | Random-zero sphygmomanometer | Mean 58.80 ± 10.04 years; 72% female | SBP: Before = 141.70 ± 7.95; After = 136.80 ± 9.10  DBP: Before = 91.60 ± 6.65; After = 87.23 ± 7.37 | Egypt |
| Ferrara et al. [46]  [BP: M(SD)] | Diet + smoking + physical activity. Thrice monthly.  MOD: face to face (group sessions) | Doctors and Dietitians | RCT; Hypertensive patients with stable blood pressure | 6 months | 188 (Control=94; Intervention=94); Random sampling | ns | Mean 56.4 ± 9.5 years; 47.87% female | SBP: Cohen’s d = -0.41 (-0.69, -0.13)  (Control = 133.1 ± 16.05; Intervention = 127.3 ± 12)  DBP: Cohen’s d = -0.18 (-0.46, 0.11)  (Control = 81.9 ± 10; Intervention = 80.3 ± 8) | Italy |
| Gabiola et al. [49]  [BP: M(SD)] | Diet + exercise; Monthly intervention  MOD: face to face (group sessions) | Resident Physicians, Nurses and Dietitians | Community trial; Pre-hypertensive and stage 1 hypertensive participants | 6 months | 159 (Control=77; Intervention = 82); Convenience sampling | ns | Median: 49.5 (40.75, 56.38); 75.4% female | SBP: Cohen’s d = -1.74 (-2.11, -1.37)  (Control = 124.28 ± 7.08; Intervention = 137.83 ± 8.46)  DBP: Cohen’s d = -1.45 (-1.80, -1.10)  (Control = 89.65 ± 2.51; Intervention = 82.91 ± 5.98) | Phillipines |
| Hazratigonbad et al. [41]  [BP: M(SD)] | Low salt diet + weight management + smoking cessation + regular physical activity; 8 sessions lasting 45 to 60 minutes each for two months  MOD: face to face (home-based) | NS | RCT; Middle-aged patients with primary HTN | 2 months | 110 (Control=55; Intervention=55); Convenience sampling | Mecury sphygmomanometer | Mean 49.46 (7.09); 71.8% female | SBP: Cohen’s d = 0.22 (-0.19, 0.63)  (Control = 124.44 ± 8.13; Intervention = 126.28 ± 8.66)  DBP: Cohen’s d = -0.02 (-0.39, 0.35)  (Control = 86.56 ± 8.51; Intervention =86.40 ± 7.96) | Iran |
| Hunt et al. [37]  [BP: M(SD)] | Healthy diet + healthy lifestyle; Intervention was delivered over the mail  MOD: mail | Primary care Physician | RCT; Patients with mildly uncontrolled HTN | 1 year (± 3 months) | 312 (Control=150; Intervention=162); Random sampling | ns | Mean 69.25 ± 12.35; 58% female | SBP: Cohen’s d = -0.13 (-0.36, 0.09)  (Control = 137 ± 15.4; Intervention = 135 ± 14.7)  DBP: Cohen’s d = 0 (-0.22, 0.22)  (Control = 77 ± 10.7; Intervention = 77 ± 11.1) | Oregon |
| Jafari et al. [42]  [BP: M(SD)] | Diet + physical activity + weight + stress management + relaxation; The intervention was implemented in 6 educational sessions during 3 weeks  MOD: face to face (group sessions) | Nurses | Clinical trial; Patients with HTN who had undergone angioplasty | 1 month | 60 (Control=30; Intervention=30); Convenient sampling method | Mecury Sphygmomanometer | Mean 57 ± 6.5 years | SBP: Cohen’s d = -0.45 (-0.81, -0.09)  (Control = 138.0 ± 15.0; Intervention = 131.7 ± 13.0)  DBP: Cohen’s d = -0.60 (-1.11, -0.08)  (Control = 83.0 ± 7.3; Intervention = 78.9 ± 6.4) | Iran |
| James et al. [50]  [BP: M(SD)] | Salt + physical exercise + smoking + abdominal obesity + weight; Intervention was done in 2 sessions for 45 minutes each  MOD: face to face (group sessions) | A post graduate trainee in Community Medicine | Non-randomized non-blinded trial; Hypertensive patients in Kamjong  block in Kamjong district, Manipur | 3 months | 66 (Control=33; Intervention=33); Convenience sampling | ns | Mean 66.06 ± 13.39 years; 57.6% female | SBP: Cohen’s d = 0.26 (-0.23, 0.74)  (Control = 143.06 ± 18.11; Intervention = 147.63 ± 17.48)  DBP: Cohen’s d = 0.14 (-0.34, 0.63)  (Control = 85.35 ± 8.97; Intervention = 86.83 ± 11.48) | India |
| Johnson et al. [38]  [BP: M(SD)] | Weight reduction + DASH eating plan + sodium & alcohol reduction + physical activity; Intervention involved 30 minutes of personal counseling by the study  nurse, at each patient visit, at 6-month intervals  MOD: face to face (group sessions) | Physicians | Randomized clinical trial; Primary care physicians and patients of  these physicians | 6 months | 552 (Patient & Physician education=249; Physician education=203; Patient education=43; No education=57); Convenience sampling | Mecury sphygmomanometer | Mean 56.75 (14.1) years; 66.05% female | PPE = SBP: 137 ± 18.6; DBP: 82 ± 12.4  PHE = SBP: 139 ± 20.3; DBP: 83 ± 13.1  PE = SBP: 143 ± 21.7; DBP: 80 ± 12.8  No Education = SBP: 139 ± 15.8; DBP = 78 ± 11.4 | USA |
| Khani Jeihooni et al. [43]  [BP: M(SD)] | Diet + smoking cessation +alcohol cessation + stress management; Intervention was carried out in ten 50–55 min sessions  MOD: face to face (group sessions with digital presentations) and via a whatsapp group | Twelve professionals in health education and health promotion,  including one nurse and one nutritionist | Randomized clinical controlled trial; Type two DM patients with HTN | 3 months | 300 (Control=150; Intervention=150); Simple random sampling | ns | Mean 53 (14) years; 55% female | SBP: Cohen’s d = -4.24 (-4.65, -3.83)  (Control = 147.04 ± 5.75; Intervention = 123.54 ± 5.32)  DBP: Cohen’s d = -7.07 (-7.68, -6.46)  (Control = 95.25 ± 3.44; Intervention = 72.24 ± 3.06) | Iran |
| Kim et al., [39]  [BP: M(SD)] | Healthy diet & nutrition + exercise; Intervention provided in 6 weekly educational sessions  MOD: face to face education & training (group sessions), BP home monitoring, and telephone counseling. | Healthcare providers including registered nurses and physicians | Clinical controlled trial; Korean-American senior participants from the Baltimore-Washington metropolitan area | 18 months | 369 (Control=185; Intervention=184); Random sampling | Digital sphygmomanometer | Mean 70.9 (5.3) years; 69.9% female | SBP: Cohen’s d = -0.17 (-0.38, 0.03)  (Control = 136 ± 19; Intervention = 133 ± 16)  DBP: Cohen’s d = -0.20 (-0.40, 0.01)  (Control = 76 ± 10; Intervention = 74 ± 10.1) | USA |
| Kordvarkane et al. [44]  [BP: M(SD)] | Extreme activity + mental & physical stress + improper diet + smoking; Training sessions were conducted individually  and face to face, once every six days, for 30-45 min each for patients. There were five sessions in total.  MOD: face to face (individually) | Nurses and a Cardiologist | RCT; Hypertensive patients who visited the heart  clinic of Farshc’s study | 3 months | 68 (Control=35; Intervention=33); Random sampling | Digital sphygmomanometer | Mean 54.53 (8.19) years; 60.83% female | SBP: Cohen’s d = -0.79 (-1.28, -0.29)  (Control = 128.62 ± 16.88; Intervention = 116.21 ± 14.52)  DBP: Cohen’s d = -0.97 (-1.47, -0.47)  (Control = 82.25 ± 9.72; Intervention = 73.93 ± 7.15) | Iran |
| Kwiringira et al. [51]  [BP: M(SD)] | Physical activity + diet; Interventions lasted for 90 minutes, every 2 weeks for 3 months  MOD: face to face (group sessions) | Community Health Workers | Quasi-experimental study; People with high blood pressure | 3 months | 2016; Random sampling | A calibrated digital automatic BP monitor | Mean 35.54 (12.33) years; 59.6% female | SBP: Before = 159 ± 29.8  After = 149 ± 29.8  DBP: Before = 97 ± 14.3  After = 92 ± 14.3 | Uganda |
| Ma et al. [40]  [BP: M(SD)] | Diet + physical activity; Intervention included a total of 2 educational sessions  MOD: face to face (group sessions) | Community health educators | RCT; Filipino participants between ages 30 and 75 years | 3 months | 71 (Control=24; Intervention=47); Random sampling | The OMRON IntelliSense® Blood Pressure  Monitor model HEM907 XL | 30-75 years; 70.5% female | SBP: Cohen’s d = -0.39 (-0.93, 0.16)  (Control = 134.80 ± 19.65; Intervention = 127.36 ± 19.01)  DBP: Cohen’s d = -0.57 (-1.07, -0.07)  (Control = 79.45 ± 8.98; Intervention = 73.33 ± 11.44) | USA |
| Thapa et al. [53]  [BP: M(SD)] | Physical activity + salt consumption + alcohol reduction + smoking cessation + stress reduction;  Intervention was received 3 times a year.  MOD: face to face (house visits) | Community Health Worker | Cluster randomized trial; Hypertensive adults aged between 25-65 years | 60 months | 395 (Control=168; Intervention=227); Cluster random sampling | HEM-7203  blood pressure monitor | Mean 50.05 (8.45) years; 59.1% female | SBP: Cohen’s d = 0.21 (0.01, 0.41)  (Control = 143.1 ± 24.1; Intervention =147.9 ± 21.9)  DBP: Cohen’s d = 0.12 (-0.08, 0.32)  (Control = 89.6 ± 13.8; Intervention = 91.1 ± 12.3) | Nepal |
