# Supplementary material for: Multi-pronged biobehavioural intervention strategies for prevention and control of hypertension: A systematic review of education-based community trials
Source: SAGE Open Med. 2026 May 10;14:20503121261444673. doi: 10.1177/20503121261444673 (PMC13168719; doi:10.1177/20503121261444673)
Supplement: sj-docx-6-smo-10.1177_20503121261444673 – Supplemental material for Multi-pronged biobehavioural intervention strategies for prevention and control of hypertension: A systematic review of education-based community trials [file sj-docx-6-smo-10.1177_20503121261444673.docx]

**Supplementary File 6: GRADE showing certainty of evidence**

| Domain | Systolic BP (SBP) | Explanation (SBP) | Diastolic BP (DBP) | Explanation (DBP) |
| --- | --- | --- | --- | --- |
| Study design | RCTs & quasi-experimental | Evidence from mixed designs, RCTs dominant | RCTs & quasi-experimental | Same structure |
| Risk of bias | Some concerns | Mostly low–moderate RoB | Some concerns | A few studies with serious concerns |
| Inconsistency | Serious | I² = 98% | Serious | I² = 99% |
| Indirectness | Not serious | Relevant population and setting | Not serious | Same justification |
| Imprecision | Some concerns | Wide prediction interval | Some concerns | Prediction interval crosses null |
| Publication bias | Not suspected | Funnel generally reassuring | Not suspected | Mixed signals; insufficient to downgrade |
| Large effect | No upgrade | Not large enough to upgrade | No upgrade | Not applicable |
| Dose-response | Not established | No clear gradient | Not established | Same |
| Overall certainty | Moderate | Downgraded for inconsistency | Low–Moderate | Downgraded for inconsistency and imprecision |
